# Subtypes in patients with opioid misuse: A prognostic enrichment strategy using electronic health record data in hospitalized patients

**DOI:** 10.1371/journal.pone.0219717

**Published:** 2019-07-16

**Authors:** Majid Afshar, Cara Joyce, Dmitriy Dligach, Brihat Sharma, Robert Kania, Meng Xie, Kristin Swope, Elizabeth Salisbury-Afshar, Niranjan S. Karnik

**Affiliations:** 1 Department of Public Health Sciences, Loyola University, Maywood, Illinois, United States of America; 2 Center for Health Outcomes and Informatics Research, Loyola University, Maywood, Illinois, United States of America; 3 Stritch School of Medicine, Loyola University, Maywood, Illinois, United States of America; 4 Department of Computer Science, Loyola University Medical Center, Maywood, Illinois, United States of America; 5 Department of Mathematics and Statistics, Loyola University, Chicago, Illinois, United States of America; 6 Center for Multi-System Solutions to the Opioid Epidemic, American Institute for Research, Chicago, Illinois, United States of America; 7 Department of Psychiatry & Behavioral Sciences, Rush University Medical Center, Chicago, Illinois, United States of America; NYU Langone Health, UNITED STATES

## Abstract

**Background:**

Approaches are needed to better delineate the continuum of opioid misuse that occurs in hospitalized patients. A prognostic enrichment strategy with latent class analysis (LCA) may facilitate treatment strategies in subtypes of opioid misuse. We aim to identify subtypes of patients with opioid misuse and examine the distinctions between the subtypes by examining patient characteristics, topic models from clinical notes, and clinical outcomes.

**Methods:**

This was an observational study of inpatient hospitalizations at a tertiary care center between 2007 and 2017. Patients with opioid misuse were identified using an operational definition applied to all inpatient encounters. LCA with eight class-defining variables from the electronic health record (EHR) was applied to identify subtypes in the cohort of patients with opioid misuse. Comparisons between subtypes were made using the following approaches: (1) descriptive statistics on patient characteristics and healthcare utilization using EHR data and census-level data; (2) topic models with natural language processing (NLP) from clinical notes; (3) association with hospital outcomes.

**Findings:**

The analysis cohort was 6,224 (2.7% of all hospitalizations) patient encounters with opioid misuse with a data corpus of 422,147 clinical notes. LCA identified four subtypes with differing patient characteristics, topics from the clinical notes, and hospital outcomes. Class 1 was categorized by high hospital utilization with known opioid-related conditions (36.5%); Class 2 included patients with illicit use, low socioeconomic status, and psychoses (12.8%); Class 3 contained patients with alcohol use disorders with complications (39.2%); and class 4 consisted of those with low hospital utilization and incidental opioid misuse (11.5%). The following hospital outcomes were the highest for each subtype when compared against the other subtypes: readmission for class 1 (13.9% vs. 10.5%, p<0.01); discharge against medical advice for class 2 (12.3% vs. 5.3%, p<0.01); and in-hospital death for classes 3 and 4 (3.2% vs. 1.9%, p<0.01).

**Conclusions:**

A 4-class latent model was the most parsimonious model that defined clinically interpretable and relevant subtypes for opioid misuse. Distinct subtypes were delineated after examining multiple domains of EHR data and applying methods in artificial intelligence. The approach with LCA and readily available class-defining substance use variables from the EHR may be applied as a prognostic enrichment strategy for targeted interventions.

## Introduction

The principles of personalized medicine to find the appropriate treatment based on a patient’s individualized determinants of health and clinical needs are a priority for improving clinical outcomes [1]. The ability to identify characteristics in patients more likely to have a clinical outcome (prognostic enrichment) is needed in conditions with a wide spectrum of clinical manifestations. In this regard, identification and treatment of opioid misuse is not a “one-size-fits-all” approach. Opioid misuse occurs along a continuum ranging from individuals who occasionally use opioids for non-medical purposes to individuals with severe opioid use disorders. The spectrum of opioid misuse impacts patients with co-occurring mental health conditions, coexisting alcohol misuse and polysubstance use, complex pain conditions, and inequities in social determinants of health [2–5]. These characteristics also influence clinical outcomes, so a tailored approach is needed to identify appropriate interventions given varying barriers to treatment for different types of misuse identified.

A data-driven approach to developing subtypes of opioid misuse using electronic health record (EHR) data has not been published in previous work. A major target group in clinical studies is patients with chronic pain and/or long-term prescription opioid use, but these targeted cohorts fail to address other types of opioid misuse behaviors that may be common in hospitalized patients [6]. Community health settings and treatment programs have used latent class analysis (LCA) from health surveys to better delineate subtypes of individuals with opioid use [7–10]. Heterogeneity in polysubstance use, illicit use, socioeconomic status, and mental illness were common subtype characteristics across study settings. Application of LCA to EHR may reveal important distinct subtypes in our patient cohort with clinically meaningful traits and demonstrate differing risks for negative health outcomes [11–13]. Identifying latent subtypes present opportunities to better align the intensity of an intervention and follow-up services for patients. The application of data-driving approaches including unsupervised learning for understanding the underlying structure of data is an important element to a learning healthcare system so that prognostic enrichment strategies are feasible from an ever-expanding quantity of EHR data.

The aim of this study is to identify subtypes of opioid misuse using readily available structured EHR data (e.g. labs, diagnoses). Additionally, the clinical notes are the largest domain of the EHR that frequently contain unstructured data (free text) about social and behavioral determinants of health that cannot be comprehensively examined manually; therefore, topic modelling was applied to summarize the corpus of text. The aim is to identify distinct subtypes of patients with opioid misuse, and provide validity with topic modelling and associations with health outcomes. We hypothesize LCA will identify distinct subtypes in our patient cohort with clinically meaningful traits and demonstrate different risks for negative health outcomes.

## Methods

### Study setting and opioid misuse definition

This study utilized data from the EHR of an urban tertiary academic center between January 1, 2007 and September 30, 2017. An operational definition for opioid misuse was developed following the National Survey on Drug Use and Health criteria for opioid misuse with criterion input from a board-certified addiction specialist (ESA) and psychiatrist (NSK). The analysis cohort included consecutive adult (≥18 years of age) emergency department and inpatient encounters meeting criteria for opioid misuse during the study period. The criteria for opioid misuse were any of the following: (1) positive urine drug screen for an opiate with polysubstance use with any of the following: an illicit drug (phencyclidine or cocaine), a benzodiazepine that is not on the patient’s medication administration record, or an amphetamine that is not on the patient’s medication administration record; (2) positive urine drug screen for an opiate but without a prescription for an opioid on the patient’s admission administration record. Urine drug screens were eligible only if no opioid or benzodiazepine drug was dispensed by the hospital pharmacy before the urine drug screen was ordered.; (3) International Classification of Diseases (ICD)-9 and –10 codes for opioid-related hospitalizations were adopted from the Healthcare Cost and Utilization Project (HCUP) [14]. ICD codes reflect final billing diagnostic codes used for claims with payers. The codes include a variety of opioid-related events and opioid misuse codes and are detailed in S1 Appendix. Many of the ICD codes do not allow for heroin-related cases to be explicitly identified. In addition, the codes do not distinguish between illegal use of prescription drugs and their use as prescribed.

To validate the operational definition, a random sample of hospital encounters was extracted from the EHR during the study period for chart review. A sample of 1,000 patient encounters including age-sex matched controls were reviewed. The annotations included an oversampling of patients who met case criteria and non-cases who had ICD codes for chronic pain, naloxone administration, or a physician order for a urine drug screen. An annotator (KS) who is an MD, MPH candidate received substance use training through Loyola’s Institute for Transformative Interprofessional Education and completed Screening, Brief Intervention, and Referral to Treatment (SBIRT) through online training. Additional training was provided to screen for likelihood of opioid misuse on a Likert Scale (1–5), and the annotator met an inter-rater reliability of a Cohen’s kappa coefficient greater than 0.80 with a critical care physician and addiction specialist (MA, ESA) before independent review was performed.

The operational definition had a sensitivity of 88.6% (95% CI 85.2%-91.9%) and specificity of 78.5% (95% CI 75.4% - 81.7%). Chart review identified many false positives that occurred in outside hospital transfers that administered an opioid during care; therefore, hospital transfers were excluded from this analysis. Cases of overdose could not be reliably discriminated using billing codes or naloxone administration with many false positives occurring as well.

Multiple encounters by the same patient were included as independent observations during analysis. As patients’ severity and subtype of misuse may change over time, our primary unit of analysis is the patient encounter in order to provide actionable insight into the subtype of misuse at hospitalization which could inform timely custom interventions. To address the potentially high correlation of intra-patient encounters, sensitivity analysis was performed to remove multiple encounters by analyzing the most recent inpatient encounter by each patient in our cohort.

### Identifying subtypes with latent class analysis (LCA)

Latent class analysis (LCA) is a statistical technique that uses mixture modelling to identify mutually exclusive and qualitatively different subgroups from multivariate categorical data [15,16]. LCA takes observed data as inputs to define a number of unobservable, distinct subtypes or classes from the population of interest. Model fit statistics are utilized to identify the appropriate number of latent classes, and the distributions of the observed class-defining indicator variables, called item response probabilities, are used to characterize the classes. Posterior probabilities for encounters indicate the likelihood of membership into each of the latent classes. The following were class-defining variables in the LCA model: (1) urine drug screen results; (2) ICD codes for opioid-related hospitalizations; (3) ICD codes for chronic pain; (4) age; (5) ICD codes for alcohol use disorders; (6) ICD codes for psychoses; (7) ICD codes for depression; and (8) ICD codes for liver disease. The eight class-defining variables for the LCA model were chosen *a priori* based on existing evidence for identifying cases of misuse and risk factors for misuse [17–21]. LCA models were considered for one to eight classes for these class-defining variables. The optimal number of latent classes was selected using fit statistics including the Bayesian information criterion (BIC), adjusted Bayesian information criterion (aBIC), consistent Akaike information criterion (cAIC), class prevalence, class separation, and model interpretability [22]. Each patient encounter was assigned a class according to the highest latent class posterior probability.

The face validity and clinical utility are examined by comparisons between latent classes using the following approaches: (1) descriptive statistics on patient characteristics and health utilization (structured EHR and census-level data) for each subtype; (2) topic models from natural language processing (clinical notes) and their probability assignment to each subtype; (3) association of subtypes with clinical outcomes (described below).

### Structured EHR and census-level data

Individual patient measures from the EHR included the following: (1) demographics and insurance status; (2) Elixhauser mortality score; (3) ICD codes for chronic pain and other disease categories developed by the Agency for Healthcare Research and Quality [23,24]; (4) hospital utilization patterns; and (5) admission service (medicine, surgery, trauma); and (6) naloxone administration (only within first three hours of first recorded vital sign). Census tract measures were used as a proxy for individual level socioeconomic status (SES). An application program interface was built to match the housing addresses to corresponding geocodes for all patients in our health system’s clinical data warehouse and provide data at the census-tract level which is equivalent to a neighborhood established by the Bureau of Census for analyzing populations. The data were collected from the 2015 American Community Survey [25] and linked to corresponding geocodes at the patient level. The census-tract measures reported for this study were the following: (1) education level (more than high school vs. high school/less than high school); (2) employment status (employed vs. unemployed); (3) median household earnings; (4) homeowner status (any homeownership vs. none); and (5) poverty level. The census-tract variable for poverty level was shown to represent an important indicator of census-level SES that correlates well with other SES measures [26,27]; therefore, we categorized patients into high- poverty census-tract (20.0+ percent of households below federal poverty level) vs. low-(≤9.9 percent of households below federal poverty level) or middle-(10.0–19.9 percent of households below federal poverty level) [28].

For identifying conditions of chronic pain [23], ICD codes for chronic primary pain, psychogenic pain, chronic postsurgical and posttraumatic pain, and chronic neuropathic pain were included. Additional codes were included for chronic secondary musculoskeletal pain and chronic secondary visceral pain. Codes were excluded for acute pain, chronic cancer-related pain, and chronic secondary headache or orofacial pain. The final list of codes is provided in S2 Appendix.

### Unstructured EHR data (clinical notes): Natural language processing and topic modelling

Pre-processing of all clinical notes was performed in the Apache clinical Text Analysis Knowledge Extraction System (cTAKES) [29]. cTAKES is a widely used software library for clinical Natural Language Processing (NLP) [30,31]. The pre-processing steps include text tokenization (splitting into words), sentence segmentation (splitting into sentences), part-of-speech tagging, and clinical concept lookup. The clinical concept lookup identified spans of clinically-relevant text, such as 'chronic pain', filtering out non-essential/non-clinical vocabulary. cTAKES maps each concept to a common standardized medical vocabulary in the Unified Medical Language System identified as a Concept Unique Identifier (CUI). This method is an approach to provide structure to otherwise unstructured text data, and the CUIs served as inputs for topic modelling.

Latent Dirichlet Allocation (LDA) is an unsupervised text mining approach used for topic modeling [32]. The LDA model was trained on the full cohort of patients with opioid misuse and produced topics expressed as a probability distribution over CUIs. LDA discovers latent topic structure by finding the mixture of CUIs that is associated with each topic and determining the probability of each topic for the individual patient encounter. We provide topic modelling for two reasons: (1) to demonstrate if the notes reflect what the administrative data and ICD codes describe for the subtypes, a representation of face validity; and (2) unstructured data from notes and reports comprise nearly 80% of the EHR data [33,34] and is a future direction in analyzing patient cohorts for clinical decision support. The optimal number of latent topics was identified by examining a range of topics with model fit statistics for topic coherence [35]. In LDA model development, 250 passes on the clinical notes were performed to learn the topics. To improve the efficiency of topic modelling, we restricted concepts, CUIs, to those observed in less than 70% and more than 10% of the EHR notes to eliminate concepts that were too commonly reported or too rare to be informative. To evaluate the contribution of a topic to an LCA-derived class, we average the probability of that topic across all the encounters in that class.

### Clinical outcomes: 30-day unplanned hospital readmission and discharge dispositions

All-cause unplanned readmission was identified using the Center for Medicare and Medicaid Services (CMS) rules for index (eligible) admission and unplanned 30-day readmission [36]. Pre-specified billing codes for planned readmission were used from CMS rules and include obstetrical delivery, scheduled procedures, maintenance chemotherapy, and rehabilitation. To analyze data on diagnoses and procedures that met qualifying criteria for readmission, the Clinical Classification Software from the Agency for Healthcare Research and Quality was used to crosswalk with diagnoses from billing codes in the EHR [37]. Readmissions during the 30-day period that follow a planned readmission are not counted in the outcome. In the case of multiple readmissions during a 30-day period, we measured only one outcome. Readmissions on the same day were also not counted in the outcome. Ultimately, index admissions include any inpatient hospitalization during the study period and are excluded for the reasons described above. Additional outcomes examined include discharge status from the hospital (in-hospital death, psychiatric admission, against medical advice (AMA), and home).

Analysis was performed using Python Version 3.6.5 (Python Software Foundation) and RStudio Version 1.1.463 (RStudio Team, Boston, MA). Latent class analysis was performed with the poLCA package in R (http://dlinzer.github.com/poLCA) and followed the analysis plan by Zhang et al. [22]. Our open-source code to perform LCA may be viewed in S3 Appendix as well as at: https://bitbucket.org/afsharjoycelab/opioid-misuse-lca/. The GenSim package was used in Python to infer topic model structure [38]. The Institutional Review Board of Loyola University Chicago approved this study.

## Results

### Opioid misuse cohort

The health system had 228,884 inpatient encounters during the study period and 6,224 (2.7%) met inclusion criteria for opioid misuse. In topic modelling, the final data corpus in the 6,224 patient encounters was comprised of 25,801 unique CUIs across 422,147 clinical notes. Twenty topics were identified to have the best model fit from the cohort (S4 Appendix). The top ten CUIs for each topic and a summarized topic theme for each topic are listed in Table 1. The topics spanned themes from chronic medical conditions to behavioral conditions and healthcare services.

**Table 1:** Topic modelling across all clinical documents in patients with opioid misuse.

Topic Number^†^	Top 10 medical terms (CUIs) within each topic (ordered highest to lowest probability within each topic)*	Topic theme (annotated from CUIs)‡
1	Kidney; Dialysis procedure; Kidney Failure, Acute; Kidney Failure; Hypertensive disease; Anemia; Pressure (finding); Chronic Kidney Diseases; Kidney Diseases; Amlodipine; Creatinine	Renal
2	Lorazepam; Thiamine; Vitamins; Vitamin B Complex; Folic Acid; Alcohol abuse; Ativan; Seizures; Multivitamin preparation; Alcohol withdrawal; Confusion	Alcohol Use Disorder intoxication/withdrawal
3	Cells; Neoplasms; Malignant neoplasms; Mass of body structure; Skeletal bone, Dilaudid; Morphine; Effusion; Biopsy; Pleural disease; Lymph node	Cancer Pain
4	Depressive disorder, Mood psychological function, Thinking function, Suicide, Axis, Suicidal, Mental State, Source, Insight, Affect (mental function)	Mental Health
5	Seizures; Magnetic Resonance Imaging; Cerebrovascular Accident; Cerebral hemisphere structure; Face; Brain; CAT scan of head; Mental state, Electroencephalography; Reflex action	Neurology (seizures)
6	Pancreatitis; Pancreas; Dilaudid; Nutrition function; Colon structure; Lipase; Nothing by mouth; Pelvis, Pantoprazole; Esophagogastroduodenoscopy	Gastrointestinal (pancreatitis)
7	Vancomycin; Microbial culture; Abscess; Blood culture; Antibiotics: Methicillin resistant Staphylococcus aureus infection; HIV infections; Bacteremia; Probe with amplification; Drainage	Infection
8	Vitamins; Warfarin; Vitamin D; Sulfate measurement; Sulfate Ion; Sulfates inorganic; Prednisone; Coumadin; Multivitamin; Calcium	Vitamin supplementation/therapy
9	Sitting position; Mobility as a finding; Independently able; Does stand; Rehabilitation therapy; Equilibrium; Physical therapy exercises; Range of motion exercises; Term birth; Supervision	Physical Therapy
10	Knee; Joint examination, Knee; Fracture; Hip structure; Procedure on Hip; Ankle; Femur; Leg; Bones of tibia; Foot	Lower extremity injury
11	Fracture; Trauma; Vertebral column; Laceration; Face; Pelvis; Skeletal bone; Neurosurgical procedures; Bone structure of rib; Hematoma	Trauma
12	Infusion procedures; Nebulizer; Vancomycin; Nutrition function; Inspiration function; Fentanyl; Albuterol; Plain Chest x-ray; Respiratory failure; Aspiration	Respiratory failure
13	Anesthesia procedure; Patient-controlled analgesia; Pyrrolidonecarboxylic Acid; Surgical incision; Drain procedure; Naloxone; Morphine; Acetaminophen; Surgical repair; Complication	Procedural pain management
14	Injury wounds; Hand; Thigh structure; Upper arm; Leg; Dressing; Dressing of wound; Body tissue; Forearm; Foot	Appendage injury
15	Liver; Hepatic; Liver cirrhosis; Ascites; Hepatitis; Transplanted tissue; Hepatoerythropoietic purpura; HEP tumour staging; Icterus; Dilated	Liver disease
16	Methadone; Heroin; Inspiration function; Asthma; Cocaine; Albuterol; Clonidine; Chronic Obstructive Airway Disease; Nebulizer solution; Hypertensive disease	Polysubstance use and inhalational complications
17	Hypertensive disease, Aspirin, Insulin, Transplanted tissue; Metoprolol; Coronary Artery Disease; Insulin Lispro; Lisinopril; Blood vessel; Lasix	Coronary artery disease
18	Integrity of skin; Impaired health; Goal achieved; Impaired skin integrity; Nursing diagnosis; Comfort alteration; At risk for falls; At risk of injury; Lack of knowledge; Impaired skin integrity	Nursing Assessment
19	Aorta; Heart ventricle; Heart atrium; Stenosis; Regurgitation; Tissue dissection; Arteries; Aortic valve structure; Veins; Kidney	Non-coronary heart
20	Human patch material; Patch-extended release; Miralax; Polyethylene; Glycol; Powder dose; Glycols; Docusate; Lidocaine; Sennosides; Fentanyl	Constipation with pain medications

† The best fit was 20 topics (coherence = 0.51) across all clinical documents (n = 422,147) with 25,801 unique CUIs.

*All entity mentions have been mapped to standardized medical vocabulary using the National Library of Medicine Unified Medical Language System Metathesaurus. The mentions are the concept unique identifier (CUI) from the free text of all the clinical documents from the EHR.

‡Overall topic themes were finalized after consensus agreement for face validity between a clinical informatics and critical care specialist, psychiatrist, and addiction specialist.

### Identification of four subtypes of opioid misuse

**Fig 1:**
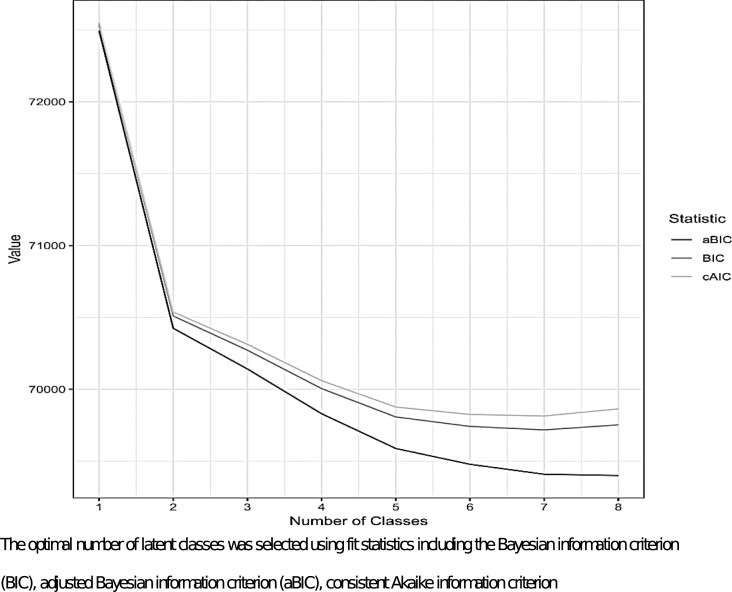
Goodness-of-fit statistics for models with varying number of classes. Caption: The optimal number of latent classes was selected using fit statistics including the Bayesian information criterion (BIC), adjusted Bayesian information criterion (aBIC), consistent Akaike information criterion.

Fit statistics and model interpretability suggest a 4-class model was optimal. Improvements to model fit begin to diminish around 5 classes (Fig 1).

Although lower model fit statistics could be achieved with more classes, the 4-class model represents meaningful and distinct clinical representation with better distribution of class prevalence and less complexity (Table 2). Details for the 5-class model are shown in S5 Appendix. In the 4-class model, the average latent class probabilities were 0.72 (sd 0.10) for Class 1, 0.87 (sd 0.12) for Class 2, and 0.88 (sd 0.11) for Class 3, and 0.95 (sd 0.12) for Class 4 indicating acceptable class separation. In addition, the 4-class model had appropriate face validity from topic modelling (Table 3). Polysubstance use as a topic had the highest probability for all classes.

**Table 2:** Patient characteristics by latent class.

	Overall (n = 6224)	Class 1: High hospital utilization with known opioid-related conditions (n = 2270)	Class 2: Illicit use, low SES, and psychoses (n = 798)	Class 3: Alcohol use disorders with complications (n = 2442)	Class 4: Low hospital utilization and incidental opioid misuse (n = 714)
Age†, n (%)					
≤ 25	413 (6.6)	234 (10.3)	43 (5.4)	58 (2.4)	78 (10.9)
26–35	1062 (17.1)	473 (20.8)	163 (20.4)	320 (13.1)	106 (14.8)
36–45	1209 (19.4)	412 (18.1)	194 (24.3)	509 (20.8)	94 (13.2)
46–55	1790 (28.8)	512 (22.6)	306 (38.3)	769 (31.5)	203 (28.4)
≥ 55	1750 (28.1)	639 (28.1)	92 (11.5)	786 (32.2)	233 (32.6)
Male, n (%)	3816 (61.3)	1153 (50.8)	472 (59.1)	1797 (73.6)	394 (55.2)
Race, n (%)					
Non-Hispanic Black	2443 (39.3)	949 (41.8)	468 (58.6)	792 (32.4)	234 (32.8)
Non-Hispanic White	3037 (48.8)	1080 (47.6)	246 (30.8)	1328 (54.4)	383 (53.6)
Hispanic	571 (9.2)	193 (8.5)	66 (8.3)	242 (9.9)	70 (9.8)
Other/Unknown	173 (2.8)	48 (2.1)	18 (2.3)	80 (3.3)	27 (3.8)
Insurance, n (%)					
Medicare	1504 (24.2)	681 (30.0)	90 (11.3)	550 (22.5)	183 (25.6)
Private	1081 (17.4)	426 (18.8)	49 (6.1)	440 (18.0)	166 (23.2)
Medicaid	2248 (36.1)	815 (35.9)	401 (50.3)	797 (32.6)	235 (32.9)
Uninsured/Other	1391 (22.3)	348 (15.3)	258 (32.3)	655 (26.8)	130 (18.2)
Elixhauser Mortality score, mean (SD)	3.7 (12.5)	1.8 (12.3)	-0.7 (9.9)	6.2 (13.0)	5.9 (11.8)
Comorbidities, n (%)					
CHF	710 (11.4)	309 (13.6)	81 (10.2)	230 (9.4)	90 (12.6)
Hypertension	3064 (49.2)	1121 (49.4)	336 (42.1)	1259 (51.6)	348 (48.7)
Neuro	1810 (29.1)	493 (21.7)	190 (23.8)	864 (35.4)	263 (36.8)
Pulmonary	1642 (26.4)	677 (29.8)	274 (34.3)	505 (20.7)	186 (26.1)
Diabetes Mellitus	759 (12.2)	281 (12.4)	67 (8.4)	308 (12.6)	103 (14.4)
Renal	714 (11.5)	317 (14.0)	66 (8.3)	266 (10.9)	65 (9.1)
Liver†	1180 (19.0)	8 (0.4)	56 (7.0)	1029 (42.1)	87 (12.2)
HIV	98 (1.6)	35 (1.5)	13 (1.6)	45 (1.8)	5 (0.7)
Alcohol use disorder†	2421 (38.9)	8 (0.4)	255 (32.0)	2052 (84.0)	106 (14.8)
Drug use*	4208 (67.6)	1861 (82.0)	732 (91.7)	1391 (57.0)	224 (31.4)
Psychoses†	1224 (19.7)	345 (15.2)	200 (25.1)	520 (21.3)	159 (22.3)
Depression†	1465 (23.5)	498 (21.9)	126 (15.8)	682 (27.9)	159 (22.3)
Chronic pain†	1829 (29.4)	728 (32.1)	226 (28.3)	596 (24.4)	279 (39.1)
Opioid misuse†	5528 (88.8)	2270 (100.0)	631 (79.1)	2442 (100)	185 (25.9)
Service, n (%)					
ER	3265 (52.5)	1059 (46.7)	479 (60.0)	1333 (54.6)	394 (55.2)
Medicine	1576 (25.3)	610 (26.9)	160 (20.1)	683 (28.0)	123 (17.2)
Trauma	495 (8.0)	93 (4.1)	112 (14.0)	158 (6.5)	132 (18.5)
Surgery	284 (4.6)	156 (6.9)	4 (0.5)	114 (4.7)	10 (1.4)
Neurology	166 (2.7)	65 (2.9)	15 (1.9)	67 (2.7)	19 (2.7)
Other	438 (7.0)	287 (12.6)	28 (3.5)	87 (3.6)	36 (5.0)
Encounters, n (%)					
Given naloxone	446 (7.2)	179 (7.9)	79 (9.9)	113 (4.6)	75 (10.5)
Given a urine drug screen	3634 (58.4)	739 (32.6)	798 (100.0)	1383 (56.6)	714 (100)
Urine drug screen (+)					
Opioids (not given / on MAR)†	903 (14.5)	176 (7.8)	280 (35.1)	91 (3.7)	356 (49.9)
Cocaine†	887 (14.3)	0 (0.0)	798 (100.0)	87 (3.6)	2 (0.3)
Phencyclidine	110 (1.8)	40 (1.8)	28 (3.5)	27 (1.1)	15 (2.1)
Benzodiazepines†	631 (10.1)	0 (0.0)	132 (16.5)	22 (0.9)	477 (66.8)
Amphetamines	107 (1.7)	25 (1.1)	17 (2.1)	19 (0.8)	46 (6.4)
Prior encounters (1 year), n (%)					
Outpatient					
0	3815 (61.3)	1092 (48.1)	654 (82.0)	1601 (65.6)	468 (65.5)
1–2	765 (12.3)	319 (14.1)	74 (9.3)	286 (11.7)	86 (12.0)
≥ 3	1644 (26.4)	859 (37.8)	70 (8.8)	555 (22.7)	160 (22.4)
Any ED	1914 (30.8)	810 (35.7)	246 (30.8)	680 (27.8)	178 (24.9)
Any IP	2433 (39.1)	1060 (46.7)	226 (28.3)	928 (38.0)	219 (30.7)
Census tract, mean (SD)					
% High Poverty	12.9 (11.4)	13.0 (11.8)	16.1 (12.3)	12.1 (10.9)	12.2 (10.7)
% Employed	38.1 (18.5)	38.4 (18.7)	37.7 (16.7)	37.9 (18.8)	38.7 (18.2)
Household earnings (Median $)	47351 (30037)	47916 (30177)	42728 (25078)	47639 (30980)	49736 (30934)
% College graduate	14.00 (9.4)	14.2 (9.6)	12.3 (8.0)	14.1 (9.5)	14.9 (9.6)
% Home owner	47.6 (23.9)	47.9 (24.0)	46.1 (20.9)	47.3 (24.5)	49.2 (24.0)

†indicates variable was included in LCA. For benzodiazepines and amphetamines, the drug was not administered during hospital encounter prior to urine drug screen testing and not on the patient’s medication administration record.

*Drug abuse codes include drug-induced mental disorder, psychoactive substances, drug-persisting dementia, polysubstance use, and use during pregnancy.

Education level = employed vs. unemployed; employment status = employed vs. unemployed; homeowner status = any homeownership vs. none; high poverty level = 20.0+ percent of household below federal poverty level.

**Table 3:** Distribution of probabilities for each topic across the 4-class latent model.

Topic	Class 1: High hospital utilization with known opioid-related conditions	Class 2: Illicit use, low SES, and psychoses	Class 3: Alcohol use disorders with complications	Class 4: Low hospital utilization and incidental opioid misuse
Polysubstance use and inhalational complications	15.2%	22.5%	7.9%	11.3%
Alcohol use disorder with intoxication/withdrawal	1.4%	5.6%	20.8%	2.5%
Gastrointestinal (pancreatitis)	10.1%	5.7%	8.0%	7.4%
Mental Health	5.7%	10.0%	7.7%	8.0%
Physical Therapy	6.8%	4.5%	5.7%	7.5%
Neurology (seizures)	5.0%	5.5%	5.8%	9.9%
Coronary artery disease	6.7%	4.6%	5.0%	6.1%
Procedural pain management	7.6%	4.4%	3.6%	6.0%
Nursing Assessment	5.8%	4.1%	5.1%	3.9%
Metastatic Cancer Pain	7.2%	3.2%	2.7%	4.1%
Trauma	2.5%	7.4%	3.4%	7.3%
Lower Extremity	4.0%	5.0%	2.4%	6.6%
Respiratory failure	3.1%	3.4%	3.9%	3.8%
Liver disease	0.9%	1.5%	6.5%	2.6%
Infection	3.7%	3.3%	2.4%	2.7%
Constipation with pain medications	3.7%	1.5%	1.6%	1.7%
Non-coronary heart disease	2.5%	2.2%	1.8%	2.4%
Appendage injury	3.0%	2.5%	1.4%	2.5%
Renal	2.4%	2.2%	1.8%	2.1%
Vitamin supplementation/therapy	2.8%	1.1%	1.8%	1.7%

Each patient encounter is assigned a distribution over all topics so each subtype/class is represented by the mean probability of the topic per subtype/class.

In sensitivity analysis examining patient-level data using the most recent hospital encounter, model fit statistics and interpretability continued to show a 4-class model to be optimal (S6 Appendix). Good class separation was found with average latent class probabilities of 0.92 (sd 0.12) for Class 1, 0.91 (sd 0.12) for Class 2, 0.77 (sd 0.11) for Class 3, and 0.94 (sd 0.14) for Class 4. The patient-level 4-class model represented nearly identical distributions of patient characteristics across demographics, comorbidities, substance use, and SES (S6 Appendix).

### Clinical distinctions between the four subtypes

The 6,224 encounters were categorized within one of the four latent classes. Class 1 represents encounters that carried a higher probability for topics on pain procedures and medical conditions associated with chronic pain (pancreatitis and metastatic cancer) (Table 3). In Table 2, approximately half were females and carry the greatest proportion with Medicare insurance among the classes. Nearly one-third had a diagnosis category for chronic pain and Class 1 had the fewest with urine drug screen testing and lowest rates for positive tests. All encounters in this class were for opioid-related hospitalizations with a greater proportion of prior 1-year inpatient and outpatient encounters compared to other classes. Class 1 comprised 36.5% of the cohort and received the label, “High hospital utilization with known opioid-related conditions”.

Class 2 are patient encounters with topics that had the highest probability for polysubstance use and mental health conditions and the greatest proportions with Elixhauser ICD codes for psychoses and drug use (Table 3). In Table 2, these patient encounters were mainly for patients 36–55 years of age and the majority were non-Hispanic black with Medicaid and uninsured status. All patient encounters in this class had positive cocaine urine drug screens. This class had the highest proportion of patients living in low socioeconomic census tracts and with the lowest median household income of all classes. Class 2 comprised 12.8% of the cohort and received the label, “Illicit use, low SES, and psychoses”.

Class 3 are patient encounters with topics that had the highest probability for alcohol use disorders and liver disease (Table 3). In Table 2, these encounters were for older and largely non-Hispanic white male patients compared to other classes, and represented the highest risk for mortality by Elixhauser score. The proportion with ICD codes for alcohol use disorders and liver disease were greatest in this class. Class 3 comprised 39.2% of the cohort and received the label, “Alcohol use disorders with complications”.

Class 4 patient encounters contained topics that had the highest probability for trauma and neurological diseases (seizures) (Table 3). In Table 2, these patients had more encounters in trauma centers with the highest proportion of urine drug testing and positive cases for opioids and benzodiazepines. Over one-third had chronic pain, similar to Class 1 but with more naloxone administration. Class 4 comprised 11.5% of the cohort and received the label, “Low hospital utilization and incidental opioid misuse”.

### Clinical outcomes between the four classes/subtypes

The class labelled as “High hospital utilization with known opioid-related conditions” (Class 1) had the greatest proportion with 30-day unplanned hospital readmission at 13.9% (Table 4). This was followed by Class 3 with the label “Alcohol use disorders with complications”. Class 2 labelled as “Illicit use, low SES, and psychoses” had the greatest proportions for being discharged to inpatient psychiatry services and leaving against medical advice. Class 4 labelled as the “Low hospital utilization with incidental opioid misuse” had the greatest proportion with naloxone administration in the hospital and in-hospital death but the lowest proportion with readmission.

**Table 4:** Latent class and clinical outcomes.

	Overall (n = 6224)	Class 1: High hospital utilizer with known opioid-related conditions (n = 2270)	Class 2: Illicit user with low SES and psychoses (n = 798)	Class 3: Alcohol use disorders with complications (n = 2442)	Class 4: Low hospital utilizer with incidental opioid misuse (n = 714)	p-value
	n (%)					
30-day unplanned readmission	725 (12.0)	309 (13.9)	67 (8.6)	292 (12.3)	57 (8.3)	<0.001
Discharge disposition						
Home	4397 (70.6)	1652 (72.8)	549 (68.8)	1717 (70.3)	479 (67.1)	<0.001
Psychiatric	314 (5.0)	67 (3.0)	53 (6.6)	155 (6.3)	39 (5.5)	
AMA	386 (6.2)	128 (5.6)	98 (12.3)	125 (5.1)	35 (4.9)	
In-hospital death	161 (2.6)	45 (2.0)	15 (1.9)	73 (3.0)	28 (3.9)	
Other	966 (15.5)	378 (16.7)	83 (10.0)	372 (15.2)	133 (18.6)	

AMA-left against medical advice; Other = long and short-term care facilities, jail, police custody

## Discussion

We identified a four-class model of clinically interpretable and relevant subtypes for opioid misuse, with good class separation and face validity based on documentation in the notes, structured data, and clinical outcomes. The following distinctions were made for each class: (1) high hospital utilization with opioid-related hospitalizations; (2) illicit use, low SES, and psychoses; (3) alcohol use disorders (AUD) with complications; (4) low hospital utilization and incidental opioid misuse. We demonstrate major differences in comorbidities, utilization patterns, polysubstance use, and SES across subtypes that may help health systems to better understand the needs of their patient population and to identify appropriate treatment options and pathways. Using an LCA approach with EHR data may better inform health systems like ours that serve diverse communities and give a level of detail that is greater than what is reported in regional or state epidemiology and surveillance data.

The subtype with the greatest proportion of patient encounters was “Alcohol use disorder with complications”. Prevalence of drug misuse is high among hospitalized patients with an AUD, but this group typically receives lower rates of treatment for addiction following hospitalization [39]. Very sparse data are available on effective treatment methods in patients with both AUD and opioid use disorder (OUD) and more research is needed. One effectiveness trial of injectable extended release naltrexone for patients with OUD found that individuals with alcohol use to intoxication in the 30 days prior to initiating treatment were more likely to relapse to opioid use in comparison to those without alcohol intoxication in the prior 30 days, indicating patients with both OUD and AUD may have worse addiction treatment outcomes [40]. In comparison to other subtypes, the patients in this subtype are older, non-Hispanic white and have higher rates of liver disease, possibly contributing to their higher risk for death. The polysubstance use and associated organ injury represent a subtype of patients that may need more intensive addiction treatment, including higher levels of care such as residential treatment.

The “high utilization with known opioid-related conditions” subtype was found to have a diverse group of patients with chronic comorbidities and pain conditions and all had an opioid-related hospitalization code. Another study identified a similar subgroup using LCA among individuals filling opioid prescriptions [8]. Many of these patients had high utilization including the greatest proportion with unplanned readmissions and nearly one-third had Medicare insurance. Multiple themes related to chronic pain conditions were present in the notes and one-third also had a billing diagnosis for chronic pain. This suggests these patients may require a treatment approach involving a comprehensive pain management team that incorporates a variety of non-pharmacologic and non-opioid alternatives and possibly reduce their need for acute care [6].

In contrast, the “Low hospital utilization and incidental opioid misuse” subtype was found to visit the trauma center more frequently and have a greater proportion of patients with positive urine drug screens for nonmedical opioid and benzodiazepine use. Naloxone was administered more frequently and this subtype had a higher proportion of in-hospital deaths. This subtype has also been previously identified using LCA for polysubstance use among trauma patients [41]. Despite positive urine drug screens, it is not possible to determine whether these patients actually have an OUD that would necessitate treatment, or whether they were occasional opioid users with subsequent trauma, injury or accidental poisoning. Screening, brief intervention, and referral to treatment (SBIRT) is one approach that could be used to identify which patients may benefit from motivational interviewing for unhealthy but infrequent use, versus others being identified with an OUD and needing initiation and/or referral to treatment [42]. Patients identified with OUD in the ED or hospital setting could be offered opioid agonist treatment, such as buprenorphine or methadone prior to discharge with linkage to community treatment. While all subtypes should be offered education on overdose prevention and naloxone prescription [43], this subtype in particular should be prioritized, as they are less likely to re-visit the health system given their low utilization pattern.

Lastly, the “Illicit use, low SES, and psychoses” subtype has the largest proportion of patient encounters with Medicaid or no insurance, suggesting patients in this subtype likely have less access to healthcare. One quarter of patients in this subtype had been diagnosed with a mental health condition in our health system, and this subtype had the highest proportion of patients leaving against medical advice. Buprenorphine initiation during the hospital encounter has been shown to reduce hospitalizations in a similar cohort of patients with heroin use [44]. The high rates of uninsured status, low SES metrics from the census-tract variables (% in poverty, unemployed, education, and median household earnings), and high rates of mental health conditions imply behavioral and social determinants of health are important considerations in this subtype [45]. These patients likely need access to intensive and comprehensive treatment programs that can offer both treatment for mental illness and for OUD (sometimes referred to as “Mental Illness and Substance Abuse” or MISA programs). Additionally, resources such as housing first models that serve individuals experiencing chronic homelessness and living with mental illness and substance use disorders may improve health-related outcomes in this subtype of patients [46,47].

Prior work examining LCA in opioid misuse were focused on self-report data in specific cohorts of individuals. One study examining approximately 200 military veterans [7] used the Overdose Risk Behavioral Scale to identify five subtypes that also revealed a “regular” opioid user category similar to our high-utilizer subtype and separate from their subtypes of occasional users and illicit users, similar to our distinctions in EHR data. In an outpatient community pharmacy study, self-report from approximately 330 surveys showed 3-classes with labels of mental health, poor health, and hazardous alcohol use [8]. This also matched our labelling of a low SES with poor mental health subtype and a co-substance alcohol subtype. In the largest studies of between 19,000 and 26,000 patients evaluated for substance use treatment programs, LCA also highlighted distinctions between polysubstance use with heroin and cocaine similar to our illicit use subtype and separate from prescription drug use subtype [9, 10]. With similarities to self-report surveys in subtypes and demographics within subtypes, EHR data may serve as another reliable source to identify clinically distinct subtypes of opioid misuse for targeted interventions.

The heterogeneity in characteristics and outcomes across subtypes present opportunities to better align intervention types and level of care/intensity of services for each subtype. Other approaches that address heterogeneity in treatment effect include adaptive treatment designs [48] as well as adaptive sampling in community health surveys [49]. Factorial experimental designs have also been used in behavioral health to better characterize individuals [50]. One example is the Sequential Multiple Assignment Randomized Trial (SMART). SMART is an approach that has been more successful than conventional treatment designs for substance use [51,52]. The results from SMART highlight the heterogeneity in treatment effect and the need for better patient identification and allocation for interventions in substance use. Herein we propose a method that caters to a health system’s patient population to identify subtypes across a cohort of individuals using LCA to augment adaptive treatment interventions like SMART [53]. This approach may be promising to identify and better address the many barriers in treating opioid misuse.

Methods in machine learning and NLP are important tools to handle the large volume and variability of EHR data. Topic modelling has been used successfully in the EHR to detect themes and relevant concepts in patient care to inform clinical decision support [54,55]. In psychiatry, similar methods have been used to predict readmissions, suicides, and accidental death with substantial improvements in model performance with LDA and other NLP methods [11,12]. However, unlike prior studies, our study first converted all the raw text into standardized medical vocabularies with CUIs to provide a common structural framework that accounts for lexical variations and semantic ambiguities. This may serve as a more interoperable approach between health systems interested in employing these methods.

Several limitations are present in this single-center study. Although we attempt to account for biases introduced in our health system with methods in NLP, topic modeling across sites may not be consistent. In addition, our operational definition for opioid misuse may introduce misclassification bias with a urine drug screen that did not capture synthetics and semi-synthetic opioids. The subtypes identified here require external validation to demonstrate if the class-defining characteristics remain consistent across multiple health systems. Bias may have been introduced by patients with multiple encounters to our health system as well as not capturing encounters at other health systems. We attempted to reduce this bias by performing sensitivity analysis of patient-level data that continued to show a 4-class model was optimal and represented the same clinical subtypes.

## Conclusion

Unsupervised statistical approaches using all domains of the EHR may be leveraged to better identify subtypes of opioid misuse in the patients served by a health system. This is a comprehensive approach to better delineate clinically meaningful subtypes so targeted treatment strategies may be employed for the patients served by the individual health system.

## Supporting information

S1 Appendix Table Operational definition algorithm including ICD 9/10 codes. (DOCX)

S2 Appendix Table ICD 9/10 codes for chronic Pain. (DOCX)

S3 Appendix Table EHR-specific R code for LCA analysis. (DOCX)

S4 Appendix Figure Coherence curve for optimal number of topics. (PNG)

S5 Appendix Table Participant characteristics by latent class for 5-class model. (DOCX)

S6 Appendix Figure/Table Coherence plot and characteristics of sensitivity analysis at patient-level. (DOCX)

## References

[1] U.S. Department of Health and Human Services Food and Drug Administration. Enrichment Strategies for Clinical Trials to Support Determination of Effectiveness of Human Drugs and Biological Products. March 2019. https://www.fda.gov/Drugs/GuidanceComplianceRegulatoryInformation/Guidelines/default.htm Accessed February 2, 2019.

[2] Barry DT, Irwin KS, Jones ES, Becker WC, Tetrault JM, Sullivan LE, et al Opioids, chronic pain, and addiction in primary care. *J Pain*. 11(12):1442–1450. 10.1016/j.jpain.2010.04.002 20627817 PMC2955997

[3] Banerjee G, Edelman EJ, Barry DT, Becker WC, Cerda M, Gaither JR, et al Non-medical use of prescription opioids is associated with heroin initiation among US veterans: a prospective cohort study. *Addiction*. 111(11):2021–2031. 10.1111/add.13491 27552496 PMC5056813

[4] Burke DS. Forecasting the opioid epidemic. *Science*. 354(6312):529 10.1126/science.aal2943 27811241

[5] Meltzer EC, Rybin D, Saitz R, Samet JH, Schwartz SL, Butler SF, et al Identifying prescription opioid use disorder in primary care: diagnostic characteristics of the Current Opioid Misuse Measure (COMM). *Pain*. 152(2):397–402. 10.1016/j.pain.2010.11.006 21177035 PMC3027065

[6] Dowell D, Haegerich TM and Chou R. CDC Guideline for Prescribing Opioids for Chronic Pain—United States, 2016. *JAMA*. 2016;315(15):1624–45. 10.1001/jama.2016.1464 26977696 PMC6390846

[7] Bennett AS, Golub A and Elliott L. A behavioral typology of opioid overdose risk behaviors among recent veterans in New York City. *PLoS One*. 2017;12:e0179054 10.1371/journal.pone.0179054 28594892 PMC5464624

[8] Cochran G, Hruschak V, Bacci JL, Hohmeier KC and Tarter R. Behavioral, mental, and physical health characteristics and opioid medication misuse among community pharmacy patients: A latent class analysis. *Res Social Adm Pharm*. 2017;13(6):1055–1061. 10.1016/j.sapharm.2016.11.005 27876595 PMC5815164

[9] Fong C, Matusow H, Cleland CM and Rosenblum A. Characteristics of Non-Opioid Substance Misusers Among Patients Enrolling in Opioid Treatment Programs: A Latent Class Analysis. *J Addict Dis*. 2015;34(2–3):141–1450. 10.1080/10550887.2015.1059226 26075932

[10] Green TC, Black R, Grimes Serrano JM, Budman SH and Butler SF. Typologies of prescription opioid use in a large sample of adults assessed for substance abuse treatment. *PLoS One*. 2011;6(11):e27244 10.1371/journal.pone.0027244 22087270 PMC3206947

[11] Chen Y, Ghosh J, Bejan CA, Gunter CA, Gypta A, Kho A, et al Building bridges across electronic health record systems through inferred phenotypic topics. *J Biomed Inform*. 2015;55:82–93. 10.1016/j.jbi.2015.03.011 25841328 PMC4464930

[12] Arnold CW, Oh A, Chen S and Speier W. Evaluating topic model interpretability from a primary care physician perspective. *Comput Methods Programs Biomed*. 2016;124:67–75. 10.1016/j.cmpb.2015.10.014 26614020 PMC4724339

[13] Huang Z, Dong W and Duan H. A probabilistic topic model for clinical risk stratification from electronic health records. *J Biomed Inform*. 2015;58:28–36. 10.1016/j.jbi.2015.09.005 26370451

[14] Compton WM, Jones CM and Baldwin GT. Relationship between Nonmedical Prescription-Opioid Use and Heroin Use. *N Engl J Med*. 2016;374(2):154–163. 10.1056/NEJMra1508490 26760086 PMC11784537

[15] Bergman LR and Magnusson D. A person-oriented approach in research on developmental psychopathology. *Dev Psychopathol*. 1997;9(2):291–319. 9201446 10.1017/s095457949700206x

[16] Collins LM. Latent class and latent transition analysis: With applications in the social, behavioral, and health sciences. New York: Wiley; 2010.

[17] Hylan TR, Von Korff M, Saunders K, Masters E, Palmer RE, Carrell D, et al Automated prediction of risk for problem opioid use in a primary care setting. *J Pain*. 2015;16(4):380–387. 10.1016/j.jpain.2015.01.011 25640294

[18] Edlund MJ, Steffick D, Hudson T, Harris KM and Sullivan M. Risk factors for clinically recognized opioid abuse and dependence among veterans using opioids for chronic non-cancer pain. *Pain*. 2007;129(3):355–362. 10.1016/j.pain.2007.02.014 17449178

[19] Sullivan MD, Edlund MJ, Zhang L, Unutzer J and Wells KB. Association between mental health disorders, problem drug use, and regular prescription opioid use. *Arch Intern Med*. 2006;166(19):2087–2093. 10.1001/archinte.166.19.2087 17060538

[20] Lee C, Sharma M, Kantorovich S and Brenton A. A Predictive Algorithm to Detect Opioid Use Disorder: What Is the Utility in a Primary Care Setting? *Health Serv Res Manag Epidemiol*. 2018;5:2333392817747467 10.1177/2333392817747467 29383324 PMC5784544

[21] Han BH, Sherman SE and Palamar JJ. Prescription opioid misuse among middle-aged and older adults in the United States, 2015–2016. *Prev Med*. 2019;121:94–98. 10.1016/j.ypmed.2019.02.018 30763631 PMC6399064

[22] Zhang Z, Abarda A, Contractor AA, Wang J and Dayton CM. Exploring heterogeneity in clinical trials with latent class analysis. *Ann Transl Med*. 2018;6(7):119 10.21037/atm.2018.01.24 29955579 PMC6015948

[23] Tonelli M, Wiebe N, Fortin M, Gurthrie B, Hemmelgarn BR, James MT et al Methods for identifying 30 chronic conditions: application to administrative data. *BMC Med Inform Decis Mak*. 2015;15:31 10.1186/s12911-015-0155-5 25886580 PMC4415341

[24] Weiss AJ, Bailey MK, O'Malley L, Barrett ML, Elixhauser A and Steiner CA. Patient Characteristics of Opioid-Related Inpatient Stays and Emergency Department Visits Nationally and by State, 2014: Statistical Brief #224 Healthcare Cost and Utilization Project (HCUP) Statistical Briefs Rockville (MD); 2006.

[25] Davern M, Quinn BC, Kenney GM and Blewett LA. The American Community Survey and health insurance coverage estimates: possibilities and challenges for health policy researchers. *Health Serv Res*. 2009;44(2 Pt 1):593–605. 10.1111/j.1475-6773.2008.00921.x 19040425 PMC2677056

[26] Krieger N, Waterman PD, Spasojevic J, Li W, Maduro G and Van Wye G. Public Health Monitoring of Privilege and Deprivation With the Index of Concentration at the Extremes. *Am J Public Health*. 2016;106(2):256–263. 10.2105/AJPH.2015.302955 26691119 PMC4815605

[27] Krieger N, Chen JT, Waterman PD, Soobader MJ, Subramanian SV and Carson R. Geocoding and monitoring of US socioeconomic inequalities in mortality and cancer incidence: does the choice of area-based measure and geographic level matter?: the Public Health Disparities Geocoding Project. *Am J Epidemiol*. 2002;156(5):471–482. 10.1093/aje/kwf068 12196317

[28] Hausauer AK, Keegan TH, Chang ET, Glaser SL, Howe H and Clarke CA. Recent trends in breast cancer incidence in US white women by county-level urban/rural and poverty status. *BMC Med*. 2009;7:31 10.1186/1741-7015-7-31 19558637 PMC2714853

[29] Savova GK, Masanz JJ, Ogren PV, Zheng J, Sohn S, Kipper-Schuler KC et al Mayo clinical Text Analysis and Knowledge Extraction System (cTAKES): architecture, component evaluation and applications. *J Am Med Inform Assoc*. 2010;17(5):507–513. 10.1136/jamia.2009.001560 20819853 PMC2995668

[30] McCoy TH Jr., Castro VM, Roberson AM, Snapper LA and Perlis RH. Improving Prediction of Suicide and Accidental Death After Discharge From General Hospitals With Natural Language Processing. *JAMA Psychiatry*. 2016;73(10):1064–1071. 10.1001/jamapsychiatry.2016.2172 27626235 PMC9980717

[31] Rumshisky A, Ghassemi M, Naumann T, Szolovits P, Castro VM, McCoy TH et al Predicting early psychiatric readmission with natural language processing of narrative discharge summaries. *Transl Psychiatry*. 2016;6(10):e921 10.1038/tp.2015.182 27754482 PMC5315537

[32] Shatte ABR, Hutchinson DM and Teague SJ. Machine learning in mental health: a scoping review of methods and applications. *Psychol Med*. 2019 49(9):1–23. 30744717 10.1017/S0033291719000151

[33] Ford E, Carroll JA, Smith HE, Scott D and Cassell JA. Extracting information from the text of electronic medical records to improve case detection: a systematic review. *J Am Med Inform Assoc*. 2016;23(5):1007–1015. 10.1093/jamia/ocv180 26911811 PMC4997034

[34] Meystre SM, Savova GK, Kipper-Schuler KC and Hurdle JF. Extracting information from textual documents in the electronic health record: a review of recent research. *Yearb Med Inform*. 2008:128–144. 18660887

[35] Roder M BA, Hinneburg A. Exploring the space of topic coherence measures. *Proceedings of the Eighth ACM International Conference on Web Search and Data Mining*. 2015.

[36] Desai NR, Ross JS, Kwon JY, Herrin J, Dharmarajan K, Bernheim SM, et al Association Between Hospital Penalty Status Under the Hospital Readmission Reduction Program and Readmission Rates for Target and Nontarget Conditions. *JAMA*. 2016;316(24):2647–2656. 10.1001/jama.2016.18533 28027367 PMC5599851

[37] Cowen ME, Dusseau DJ, Toth BG, Guisinger C, Zodet MW and Shyr Y. Casemix adjustment of managed care claims data using the clinical classification for health policy research method. *Med Care*. 1998;36(7):1108–1013. 9674627 10.1097/00005650-199807000-00016

[38] Rehurek R SP. Software framework for topic modelling with large corpora. *Proc LR 2010 Work New Challenges NLP fram* 2010:45–50.

[39] Smothers BA and Yahr HT. Alcohol use disorder and illicit drug use in admissions to general hospitals in the United States. *Am J Addict*. 2005;14(3):256–267. 10.1080/10550490590949433 16019976

[40] Friedmann PD, Wilson D, Nunes EV, Hoskinson R Jr., Lee JD, Gordon M, et al Do patient characteristics moderate the effect of extended-release naltrexone (XR-NTX) for opioid use disorder? *J Subst Abuse Treat*. 2018;85:61–65. 10.1016/j.jsat.2017.01.018 28236511 PMC5565721

[41] Scherer M, Romano E, Voas R and Taylor E. Latent Classes of Polydrug Users as a Predictor of Crash Involvement and Alcohol Consumption. *J Stud Alcohol Drugs*. 2018;79(3):481–489. 10.15288/jsad.2018.79.481 29885157 PMC6005252

[42] Thompson H HK, Jadhav R, Webb TA, Pollack M, Karnik N. The subtance use intervention team: A preliminary analysis of a population-level strategy to address the opioid crisis at an academic health center. *J Addict Med*. 2019;Epub ahead of print. 10.1097/ADM.0000000000000520 PMC6844647 31689260

[43] Jeffery RM, Dickinson L, Ng ND, DeGeorge LM and Nable JV. Naloxone administration for suspected opioid overdose: An expanded scope of practice by a basic life support collegiate-based emergency medical services agency. *J Am Coll Health*. 2017;65(3):212–216. 10.1080/07448481.2016.1277730 28059635

[44] Moreno JL, Wakeman SE, Duprey MS, Roberts RJ, Jacobson JS and Devlin JW. Predictors for 30-Day and 90-Day Hospital Readmission Among Patients With Opioid Use Disorder. *J Addict Med*. 2019 [Epub ahead of print]. 10.1097/ADM.0000000000000499 30633044

[45] Pytell JD and Rastegar DA. Who Leaves Early? Factors Associated With Against Medical Advice Discharge During Alcohol Withdrawal Treatment. *J Addict Med*. 2018;12(6):447–452. 10.1097/ADM.0000000000000430 29939873

[46] Watson DP, Shuman V, Kowalsky J, Golembiewski E and Brown M. Housing First and harm reduction: a rapid review and document analysis of the US and Canadian open-access literature. *Harm Reduct J*. 2017;14(1):30 10.1186/s12954-017-0158-x 28535804 PMC5442650

[47] Fitzpatrick-Lewis D, Ganann R, Krishnaratne S, Ciliska D, Kouyoumdjian F and Hwang SW. Effectiveness of interventions to improve the health and housing status of homeless people: a rapid systematic review. *BMC public health*. 2011;11:638 10.1186/1471-2458-11-638 21831318 PMC3171371

[48] Murphy SA, Lynch KG, Oslin D, McKay JR and TenHave T. Developing adaptive treatment strategies in substance abuse research. *Drug Alcohol Depend*. 2007;88 Suppl 2:S24–30. 17056207 10.1016/j.drugalcdep.2006.09.008 PMC1922034

[49] Thompson SK and Collins LM. Adaptive sampling in research on risk-related behaviors. *Drug Alcohol Depend*. 2002;68 Suppl 1:S57–67. 12324175 10.1016/s0376-8716(02)00215-6

[50] Piper ME, Schlam TR, Cook JW, Smith SS, Bolt DM, Loh WY, et al Toward precision smoking cessation treatment I: Moderator results from a factorial experiment. *Drug Alcohol Depend*. 2017;171:59–65. 10.1016/j.drugalcdep.2016.11.025 28013098 PMC5263119

[51] Lei H, Nahum-Shani I, Lynch K, Oslin D and Murphy SA. A "SMART" design for building individualized treatment sequences. *Annu Rev Clin Psychol*. 2012;8:21–48. 10.1146/annurev-clinpsy-032511-143152 22224838 PMC3887122

[52] Nahum-Shani I, Qian M, Almirall D, Pelham WE, Gnagy B, Fabiano GA, et al Experimental design and primary data analysis methods for comparing adaptive interventions. *Psychol Methods*. 2012;17:457–477. 10.1037/a0029372 23025433 PMC3825557

[53] Hay KR, Huhn AS, Tompkins DA and Dunn KE. Recovery Goals and Long-term Treatment Preference in Persons Who Engage in Nonmedical Opioid Use. *J Addict Med*. 2019. [Epub ahead of print] 10.1097/ADM.0000000000000498 PMC6609500 30633045

[54] Chen JH, Goldstein MK, Asch SM, Mackey L and Altman RB. Predicting inpatient clinical order patterns with probabilistic topic models vs conventional order sets. *J Am Med Inform Assoc*. 2017;24(3):472–480. 10.1093/jamia/ocw136 27655861 PMC5391730

[55] Huang Z, Ge Z, Dong W, He K and Duan H. Probabilistic modeling personalized treatment pathways using electronic health records. *J Biomed Inform*. 2018;86:33–48. 10.1016/j.jbi.2018.08.004 30138699

