# Supplementary material for: Subtypes in patients with opioid misuse: A prognostic enrichment strategy using electronic health record data in hospitalized patients
Source: PLoS One. 2019 Jul 16;14(7):e0219717. doi: 10.1371/journal.pone.0219717 (PMC6634397; doi:10.1371/journal.pone.0219717)
Supplement: S1 Appendix Table — (DOCX) [file pone.0219717.s001.docx]

**Supplemental 1. Operational Definition algorithm including ICD 9/10 codes**

**I.** First urine toxicology (+) for opioid only and no evidence of opioid on medication administration record

OR

First urine toxicology (+) for opioid (regardless of medication list) and also (+) urine toxicology screen for any the following:

(1) benzodiazepine and no evidence of benzodiazepine on medication administration record;

(2) amphetamine and no evidence of amphetamine on medication administration record;

(3) phencyclidine;

(4) cocaine

AND

No opioid or benzodiazepine dispensed before urine toxicology result timestamp of the encounter

**Loyola urine toxicology assay used in the emergency department and hospital (not outpatient/pain clinics) does not detect methadone, oxycodone, oxymorphone, fentanyl, buprenorphine, meperidine, tramadol, and any other synthetics/semi-synthetics*

**AND/OR**

**II.** *ICD-9-CM Codes Prior to October 1, 2015* and *ICD-10-CM Codes Starting October 1, 2015* (adopted from AHRQ’s Healthcare and Cost Utilization Project)

Codes for opioid abuse

| **ICD-9-CM Code** | **ICD-10-CM Code** | **Description** |
| --- | --- | --- |
| 30550 |  | Opioid abuse, unspecified |
| 30551 |  | Opioid abuse, continuous |
| 30552 |  | Opioid abuse, episodic |
|  | F1110 | Opioid abuse, uncomplicated |
|  | F11120 | Opioid abuse with intoxication, uncomplicated |
|  | F11121 | Opioid abuse with intoxication delirium |
|  | F11122 | Opioid abuse with intoxication with perceptual disturbance |
|  | F11129 | Opioid abuse with intoxication, unspecified |
|  | F1114 | Opioid abuse with opioid-induced mood disorder |
|  | F11150 | Opioid abuse with opioid-induced psychotic disorder with delusions |
|  | F11151 | Opioid abuse with opioid-induced psychotic disorder with hallucinations |
|  | F11159 | Opioid abuse with opioid-induced psychotic disorder, unspecified |
|  | F11181 | Opioid abuse with opioid-induced sexual dysfunction |
|  | F11182 | Opioid abuse with opioid-induced sleep disorder |
|  | F11188 | Opioid abuse with other opioid-induced disorder |
|  | F1119 | Opioid abuse with unspecified opioid-induced disorder |

Codes for opioid dependence

| **ICD-9-CM Code** | **ICD-10-CM Code** | **Description** |
| --- | --- | --- |
| 30400 |  | Opioid type dependence, unspecified |
| 30401 |  | Opioid type dependence, continuous |
| 30402 |  | Opioid type dependence, episodic |
| 30470 |  | Combinations of opioid type drug with any other drug dependence, unspecified |
| 30471 |  | Combinations of opioid type drug with any other drug dependence, continuous |
| 30472 |  | Combinations of opioid type drug with any other drug dependence, episodic |
|  | F1120 | Opioid dependence, uncomplicated |
|  | F11220 | Opioid dependence with intoxication, uncomplicated |
|  | F11221 | Opioid dependence with intoxication delirium |
|  | F11222 | Opioid dependence with intoxication with perceptual disturbance |
|  | F11229 | Opioid dependence with intoxication, unspecified |
|  | F1123 | Opioid dependence with withdrawal |
|  | F1124 | Opioid dependence with opioid-induced mood disorder |
|  | F11250 | Opioid dependence with opioid-induced psychotic disorder with delusions |
|  | F11251 | Opioid dependence with opioid-induced psychotic disorder with hallucinations |
|  | F11259 | Opioid dependence with opioid-induced psychotic disorder, unspecified |
|  | F11281 | Opioid dependence with opioid-induced sexual dysfunction |
|  | F11282 | Opioid dependence with opioid-induced sleep disorder |
|  | F11288 | Opioid dependence with other opioid-induced disorder |
|  | F1129 | Opioid dependence with unspecified opioid-induced disorder |

Codes for adverse effects of opioids

| **ICD-9-CM Code** | **ICD-10-CM Code** | **Description** |
| --- | --- | --- |
| **Adverse Effects of Opioids** | | |
| E9350 |  | Heroin causing adverse effects in therapeutic use |
| E9351 |  | Methadone causing adverse effects in therapeutic use |
| E9352 |  | Other opiates and related narcotics causing adverse effects in therapeutic use |
| E9401 |  | Adverse effects of opiate antagonists |
|  | T400X5A | Adverse effect of opium, initial encounter |
|  | T400X5D | Adverse effect of opium, subsequent encounter |
|  | T400X5S | Adverse effect of opium, sequela |
|  | T402X5A | Adverse effect of other opioids, initial encounter |
|  | T402X5D | Adverse effect of other opioids, subsequent encounter |
|  | T402X5S | Adverse effect of other opioids, sequela |
|  | T403X5A | Adverse effect of methadone, initial encounter |
|  | T403X5D | Adverse effect of methadone, subsequent encounter |
|  | T403X5S | Adverse effect of methadone, sequela |
|  | T404X5A | Adverse effect of synthetic narcotics, initial encounter |
|  | T404X5D | Adverse effect of synthetic narcotic, subsequent encounter |
|  | T404X5S | Adverse effect of synthetic narcotic, sequela |
|  | T40605A | Adverse effect of unspecified narcotics, initial encounter |
|  | T40605D | Adverse effect of unspecified narcotics, subsequent encounter |
|  | T40605S | Adverse effect of unspecified narcotics, sequela |
|  | T40695A | Adverse effect of other narcotics initial encounter |
|  | T40695D | Adverse effect of other narcotics, subsequent encounter |
|  | T40695S | Adverse effect of other narcotics, sequela |

Codes for opioid poisoning

| **ICD-9-CM Code** | **ICD-10-CM Code** | **Description** |
| --- | --- | --- |
| 96500 |  | Poisoning by opium (alkaloids), unspecified |
| 96501 |  | Poisoning by heroin |
| 96502 |  | Poisoning by methadone |
| 96509 |  | Poisoning by other opiates and related narcotics |
| 9701 |  | Poisoning by opiate antagonists |
| E8500 |  | Accidental poisoning by heroin |
| E8501 |  | Accidental poisoning by methadone |
| E8502 |  | Accidental poisoning by other opiates and related narcotics |
|  | T400X1A | Poisoning by opium, accidental (unintentional), initial encounter |
|  | T400X1D | Poisoning by opium, accidental (unintentional), subsequent encounter |
|  | T400X1S | Poisoning by opium, accidental (unintentional), sequela |
|  | T400X4A | Poisoning by opium, undetermined, initial encounter |
|  | T400X4D | Poisoning by opium, undetermined, subsequent encounter |
|  | T400X4S | Poisoning by opium, undetermined, sequela |
|  | T401X1A | Poisoning by heroin, accidental (unintentional), initial encounter |
|  | T401X1D | Poisoning by heroin, accidental (unintentional), subsequent encounter |
|  | T401X1S | Poisoning by heroin, accidental (unintentional), sequela |
|  | T401X4A | Poisoning by heroin, undetermined, initial encounter |
|  | T401X4D | Poisoning by heroin, undetermined, subsequent encounter |
|  | T401X4S | Poisoning by heroin, undetermined, sequela |
|  | T402X1A | Poisoning by other opioids, accidental (unintentional), initial encounter |
|  | T402X1D | Poisoning by other opioids, accidental (unintentional), subsequent encounter |
|  | T402X1S | Poisoning by other opioids, accidental (unintentional), sequela |
|  | T402X4A | Poisoning by other opioids, undetermined, initial encounter |
|  | T402X4D | Poisoning by other opioids, undetermined, subsequent encounter |
|  | T402X4S | Poisoning by other opioids, undetermined, sequela |
|  | T403X1A | Poisoning by methadone, accidental (unintentional), initial encounter |
|  | T403X1D | Poisoning by methadone, accidental (unintentional), subsequent encounter |
|  | T403X1S | Poisoning by methadone, accidental (unintentional), sequela |
|  | T403X4A | Poisoning by methadone, undetermined, initial encounter |
|  | T403X4D | Poisoning by methadone, undetermined, subsequent encounter |
|  | T403X4S | Poisoning by methadone, undetermined, sequela |
|  | T404X1A | Poisoning by synthetic narcotics, accidental (unintentional), initial encounter |
|  | T404X1D | Poisoning by synthetic narcotics, accidental (unintentional), subsequent encounter |
|  | T404X1S | Poisoning by synthetic narcotics, accidental (unintentional), sequela |
|  | T404X4A | Poisoning by synthetic narcotics, undetermined, initial encounter |
|  | T404X4D | Poisoning by synthetic narcotics, undetermined, subsequent encounter |
|  | T404X4S | Poisoning by synthetic narcotics, undetermined, sequela |
|  | T40601A | Poisoning by unspecified narcotics, accidental (unintentional), initial encounter |
|  | T40601D | Poisoning by unspecified narcotics, accidental (unintentional), subsequent encounter |
|  | T40601S | Poisoning by unspecified narcotics, accidental (unintentional), sequela |
|  | T40604A | Poisoning by unspecified narcotics, undetermined, initial encounter |
|  | T40604D | Poisoning by unspecified narcotics, undetermined, subsequent encounter |
|  | T40604S | Poisoning by unspecified narcotics, undetermined, sequela |
|  | T40691A | Poisoning by other narcotics, accidental (unintentional), initial encounter |
|  | T40691D | Poisoning by other narcotics, accidental (unintentional), subsequent encounter |
|  | T40691S | Poisoning by other narcotics, accidental (unintentional), sequela |
|  | T40694A | Poisoning by other narcotics, undetermined, initial encounter |
|  | T40694D | Poisoning by other narcotics, undetermined, subsequent encounter |
|  | T40694S | Poisoning by other narcotics, undetermined, sequela |

Codes for opioid use, unspecified (There are no ICD-9-CM equivalents for these codes)

| **ICD-10-CM Code** | **Description** |
| --- | --- |
| F1190 | Opioid use, unspecified, uncomplicated |
| F11920 | Opioid use, unspecified, with intoxication, uncomplicated |
| F11921 | Opioid use, unspecified, with intoxication delirium |
| F11922 | Opioid use, unspecified, with intoxication with perceptual disturbance |
| F11929 | Opioid use, unspecified, with intoxication, unspecified |
| F1193 | Opioid use, unspecified with withdrawal |
| F1194 | Opioid use, unspecified with opioid-induced mood disorder |
| F11950 | Opioid use, unspecified with opioid-induced psychotic disorder with delusions |
| F11951 | Opioid use, unspecified with opioid-induced psychotic disorder with hallucinations |
| F11959 | Opioid use, unspecified with opioid-induced psychotic disorder, unspecified |
| F11981 | Opioid use, unspecified with opioid-induced sexual dysfunction |
| F11982 | Opioid use, unspecified with opioid-induced sleep disorder |
| F11988 | Opioid use, unspecified with other opioid-induced disorder |
| F1199 | Opioid use, unspecified with unspecified opioid-induced disorder |
