# Supplementary material for: Subtypes in patients with opioid misuse: A prognostic enrichment strategy using electronic health record data in hospitalized patients
Source: PLoS One. 2019 Jul 16;14(7):e0219717. doi: 10.1371/journal.pone.0219717 (PMC6634397; doi:10.1371/journal.pone.0219717)
Supplement: S2 Appendix Table — (DOCX) [file pone.0219717.s002.docx]

**Supplemental 2. ICD 9/10 codes for chronic Pain**

ICD 9/10 for chronic primary pain, psychogenic pain, chronic postsurgical and posttraumatic pain, chronic neuropathic pain, chronic secondary musculoskeletal pain, chronic secondary visceral pain (inflammation).

EXCLUDES acute pain, chronic cancer-related pain, chronic secondary headache or orofacial pain,

'338', '338.0', '338.2', '338.21', '338.22', '307.80', '307.89', '719.41', '719.45', '719.46', '719.47', '338.28', '338.29', '338.4', '729.1', '724.5', '719.48', '719.49', '720.0', '720.2', '720.9', '721.0', '721.1', '721.2', '721.3', '721.4', '721.6', '721.8', '721.9', '722', '723.0', '723.1', '723.3', '723.4', '723.5', '723.6', '723.7', '723.8', '723.9', '724.0', '724.1','724.2', '724.3', '724.4', '724.5', '724.6', '724.70', '724.79', '724.8', '724.9', '729.0', '729.1', '729.2', '729.4', '729.5', 'F45.4', 'G89', 'G89.0', 'G89.2', 'G89.21', 'G89.22', 'G89.28', 'G89.29', 'G89.4', 'M08.1', 'M25.50', 'M25.51', 'M25.55', 'M25.56', 'M25.57', 'M43.2', 'M43.3', 'M43.4', 'M43.5', 'M43.6', 'M45', 'M46.1', 'M46.3', 'M46.4', 'M46.9', 'M47', 'M48.0', 'M48.1', 'M48.8', 'M48.9', 'M50.8', 'M50.9', 'M51', 'M53.1', 'M53.2', 'M53.3', 'M53.8', 'M53.9', 'M54', 'M60.8', 'M60.9', 'M63.3', 'M79.0', 'M79.1', 'M79.2', 'M79.6', 'M79.7', 'M96.1'
