# Supplementary material for: Subtypes in patients with opioid misuse: A prognostic enrichment strategy using electronic health record data in hospitalized patients
Source: PLoS One. 2019 Jul 16;14(7):e0219717. doi: 10.1371/journal.pone.0219717 (PMC6634397; doi:10.1371/journal.pone.0219717)
Supplement: S3 Appendix Table — (DOCX) [file pone.0219717.s003.docx]

#install.packages("poLCA")

#install.packages("gmodels")

#install.packages("tableone")

library(poLCA)

library(ggplot2)

library(gmodels)

library(tableone)

library(dplyr)

library(tidyr)

library(readr)

library(lubridate)

# Updated analytic dataset (n=7086 **with** opioid misuse)

load(file = “”)

#########################################################

#########################################################

### Data preparation **for** LCA ###

### Note: input variables must be integers ###

### starting at 1 ###

### Will want original variables **for** Table1 ###

#########################################################

#########################################################

adt <- read.delim("/home/shared/PROJECTS/03_OpioidPhenotype/DATA/uc_adt.txt", head = T, sep = "|", fill = T)

emptycols <- colSums(**is**.na(adt)) == nrow(adt)

adt <- adt[!emptycols]

table(adt$pat_service)

burn_adt = adt %>% filter(pat_service == 'BURN') %>% distinct(hsp_account_id) %>% mutate(burn = 1)

transfers = read.delim("/home/shared/RAW-DATA/RAW_PHI/01_ENCOUNTERS/transfers.csv", head = T, sep = ",", fill = T)

transfers = transfers %>% select(hsp_account_id, transferred_from) %>%

mutate(hsp_account_id = **as**.numeric(**as**.character(hsp_account_id)))

summary(transfers$hsp_account_id)

for_lca = subset(op_analytic, flag_pos_opioids == 1)

for_lca = left_join(for_lca, burn_adt, by = "hsp_account_id") %>% filter(**is**.na(burn))

for_lca = left_join(for_lca, transfers, by = "hsp_account_id") %>% mutate(flag_transfer = !**is**.na(transferred_from))

for_lca = for_lca %>% filter(flag_transfer == 0 | (flag_transfer == 1 & flag_dx_opioid == 1))

for_lca$cat_race = ifelse((for_lca$race == "Black"),"Black",

ifelse((for_lca$race == "White"),"White",

ifelse(for_lca$race != "Unknown" & for_lca$face != "", "Other", "Unknown")))

for_lca$raceeth = ifelse((for_lca$race == "Black" & for_lca$ethnic == "Non-Hispanic Origin"),1,

ifelse((for_lca$race == "White" & for_lca$ethnic == "Non-Hispanic Origin"),2,

ifelse(for_lca$ethnic == "Hispanic Origin", 3, 4)))

for_lca$sex = ifelse(for_lca$gender == 'F', 1, ifelse(for_lca$gender == 'M', 2, NA))

for_lca$cat_ins = ifelse(for_lca$insurance == 'Private', 1,

ifelse(for_lca$insurance == 'Medicare', 2, 3))

for_lca$count_ed365 = **as**.numeric(for_lca$count_ed365)

for_lca$count_op365 = **as**.numeric(for_lca$count_op365)

for_lca$count_op30 = **as**.numeric(for_lca$count_op30)

for_lca$cat_ed365 = ifelse(for_lca$count_ed365 > 0, 1, 0)

#for_lca$cat_ip365 = ifelse(for_lca$count_ip365 > 0, 1, 0)

for_lca$cat_op365 = ifelse(for_lca$count_op365 >= 3, 3,

ifelse(for_lca$count_op365 >= 1, 2, 1))

for_lca$cat_ip365 = ifelse(for_lca$count_ip365 >= 3, 3,

ifelse(for_lca$count_ip365 >= 1, 2, 1))

for_lca$flag_chronpain = for_lca$flag_dx_chronpain

for_lca$flag_opioid = for_lca$flag_dx_opioid

for_lca$flag_dx_chronpain = ifelse(for_lca$flag_dx_chronpain == 0, 2, for_lca$flag_dx_chronpain)

for_lca$flag_dx_opioid = ifelse(for_lca$flag_dx_opioid == 0, 2, for_lca$flag_dx_opioid)

#for_lca$flag_given_narcan = ifelse(for_lca$flag_given_narcan == 0, 2, for_lca$flag_given_narcan)

#for_lca$flag_ds_opioids = ifelse(for_lca$flag_ds_opioids == 0, 2, for_lca$flag_ds_opioids)

for_lca$flag_drugscreen_opioids = ifelse(for_lca$flag_ds_noprescribe_opioids == 0, 2, for_lca$flag_ds_noprescribe_opioids)

for_lca$flag_drugscreen_cocaine = ifelse(for_lca$flag_ds_pos_cocaine == 0, 2, for_lca$flag_ds_pos_cocaine)

#for_lca$flag_ds_pos_phencyc = ifelse(for_lca$flag_ds_pos_phencyc == 0, 2, for_lca$flag_ds_pos_phencyc)

for_lca$flag_drugscreen_benzo = ifelse(for_lca$flag_ds_noprescribe_benzo == 0, 2, for_lca$flag_ds_noprescribe_benzo)

for_lca$flag_drugscreen_pos_amphet = ifelse(for_lca$flag_ds_noprescribe_amphet == 0, 2, for_lca$flag_ds_noprescribe_amphet)

for_lca$age = ifelse(for_lca$pt_age <= 25, 1, ifelse(for_lca$pt_age <= 35, 2,

ifelse(for_lca$pt_age <= 45, 3,

ifelse(for_lca$pt_age <= 55, 4, 5))))

for_lca$flag_alcohol = for_lca$Alcohol

for_lca$flag_drugs = for_lca$Drugs

for_lca$flag_psychoses = for_lca$Psychoses

for_lca$flag_depression = for_lca$Depression

for_lca$flag_neuro = for_lca$NeuroOther

for_lca$flag_liver = for_lca$Liver

for_lca$flag_obesity = for_lca$Obesity

for_lca$Alcohol = ifelse(for_lca$Alcohol == 0, 2, for_lca$Alcohol)

for_lca$Drugs = ifelse(for_lca$Drugs == 0, 2, for_lca$Drugs)

for_lca$Psychoses = ifelse(for_lca$Psychoses == 0, 2, for_lca$Psychoses)

for_lca$Depression = ifelse(for_lca$Depression == 0, 2, for_lca$Depression)

for_lca$NeuroOther = ifelse(for_lca$NeuroOther == 0, 2, for_lca$NeuroOther)

for_lca$Liver = ifelse(for_lca$Liver == 0, 2, for_lca$Liver)

for_lca$Obesity = ifelse(for_lca$Obesity == 0, 2, for_lca$Obesity)

for_lca$flag_op30 <- ifelse(for_lca$count_op30 > 0, 1, 0)

summary(for_lca)

#CrossTable(for_lca$flag_ds_opioids, for_lca$flag_ds_pos_opioids)

#CrossTable(**as**.numeric(analytic$count_ed365), analytic$flag_pos_opioids, format="SAS")

#CrossTable(**as**.numeric(analytic$count_op365), analytic$flag_pos_opioids, format="SAS")

#CrossTable(**as**.numeric(analytic$count_ed365), analytic$flag_pos_opioids, format="SAS")

for_lca = for_lca %>% mutate(rownum = row_number())

#########################################################

#########################################################

### Run LCA models **for** varying **number** **of** classes ###

### Note: added nrep here ###

### Number **of** times to estimate the model, ###

### using different values **of** probs.start. ###

### The **default** **is** one. Setting nrep>1 ###

### automates the search **for** the **global** — ###

### rather than just a local—maximum **of** the ###

### log-likelihood **function**. poLCA returns ###

### the parameter estimates corresponding ###

### to the model **with** the greatest ###

### log-likelihood ###

#########################################################

#########################################################

**for**(i **in** 1:8){

assign(paste("lc",i,sep=""),

poLCA(cbind(age, Alcohol, Psychoses, Depression, Liver, flag_dx_opioid, flag_dx_chronpain, flag_drugscreen_opioids,

flag_drugscreen_cocaine, flag_drugscreen_benzo)~1, data=for_lca, nclass=i, maxiter=50000,

tol=1e-5, na.rm=FALSE,

nrep=10, verbose=TRUE, calc.se=TRUE))

}

#########################################################

#########################################################

### Create table **of** fit statistics and figures ###

### Modeled on: ###

### http:*//dx.doi.org/10.21037/atm.2018.01.24 ###*

#########################################################

#########################################################

fitstats = data.frame(matrix(rep(999,8),nrow=1))

names(fitstats)<-c("Log Likelihood", "df","BIC", "aBIC","cAIC","Likelihood Ratio","Entropy")

entropy = **function**(lc){

**return**(-sum(lc$posterior*log(lc$posterior),

na.rm=T))

}

relative.entropy= **function**(lc){

en<--sum(lc$posterior*

log(lc$posterior),na.rm=T)

e<-1-en/(nrow(lc$posterior)*log(ncol(lc$posterior)))

**return**(e)

}

**for**(i **in** 1:8){

fitstats = rbind(fitstats,

c(**get**(paste("lc",i,sep=""))$llik,

**get**(paste("lc",i,sep=""))$resid.df,

**get**(paste("lc",i,sep=""))$bic,

(-2***get**(paste("lc",i,sep=""))$llik) +

((log((**get**(paste("lc",i,sep=""))$N + 2)/24)) *

**get**(paste("lc",i,sep=""))$npar),

(-2***get**(paste("lc",i,sep=""))$llik) +

**get**(paste("lc",i,sep=""))$npar *

(1 + log(**get**(paste("lc",i,sep=""))$N)),

**get**(paste("lc",i,sep=""))$Gsq,

relative.entropy(**get**(paste("lc",i,sep="")))

))

}

fitstats = round(fitstats[-1,],2)

fitstats$Classes = 1:8

fit_long = fitstats %>% gather(key = "Statistic", value = "Value", 1:7)

fit_long = fit_long[,2:4]

fit_long %>%

filter(Statistic != 'df' & Statistic != 'Log Likelihood' & Classes > 1) %>%

ggplot(aes(x = Classes, y = Value)) + geom_line() +

facet_grid(Statistic ~ ., scales = "free") +

xlab("Number of Classes") + ylab("Fit Statistic") + scale_x_continuous(breaks = c(1:8)) + theme_bw()

fit_long %>%

filter(Statistic == 'BIC' | Statistic == 'aBIC' | Statistic == 'cAIC') %>%

ggplot(aes(x = Classes, y = Value, fill = Statistic, col = Statistic)) + geom_line() +

xlab("Number of Classes") + scale_x_continuous(breaks = c(1:8)) + theme_bw() + scale_colour_grey()

#########################################################

### Variables to score LCA models on original dataset ###

#########################################################

post = for_lca %>%

dplyr::select(age, Alcohol, Psychoses, Depression, Liver, flag_dx_opioid, flag_dx_chronpain, flag_drugscreen_opioids,

flag_drugscreen_cocaine, flag_drugscreen_benzo)

catvars = c("age", "gender", "cat_race", "raceeth", "insurance",

"CHF", "Valvular", "PHTN", "PVD",

"HTN", "Paralysis", "flag_neuro", "Pulmonary", "DM", "DMcx",

"Hypothyroid", "Renal", "flag_liver", "PUD", "HIV", "Lymphoma", "Mets",

"Tumor", "Rheumatic", "Coagulopathy", "flag_obesity", "WeightLoss",

"FluidsLytes", "BloodLoss", "Anemia", "flag_alcohol", "flag_drugs", "flag_psychoses",

"flag_depression", "max_pain", "service_cat", "flag_chronpain", "flag_opioid",

"flag_given_narcan", "flag_test_urinedrug", "flag_ds_opioids",

"flag_ds_pos_opioids", "flag_ds_pos_cocaine", "flag_ds_pos_phencyc",

"flag_ds_pos_benzo", "flag_ds_pos_amphet",

"flag_ds_noprescribe_opioids", "flag_ds_noprescribe_benzo", "flag_ds_noprescribe_amphet",

"cat_ip365", "cat_op365", "cat_ed365")

contvars = c("pt_age", "elix_readmit", "elix_mortality", "count_ed30", "count_ip30",

"count_op30", "count_ed365", "count_ip365", "count_op365", "PropMarried", "PropYrs85Plus",

"PropBlack", "PropYrs519", "PerCapitaIncome", "PropNhWhite",

"PropNhBlack", "PropUnemployed", "PropHBlack", "PropCollegeGrad",

"PropPoverty", "PropAsian", "PropHighSchool", "PropFoodStamps",

"PropWhite", "PropHispanic", "PropYrs3544", "PropYrs5564", "PropYrs2534",

"PropDisabled", "PropNeverMarried", "PropYrsUnder5", "PropSomeCollege",

"PropYrs2024", "PropEmployed", "PropYrs7584", "MedianEarnings",

"PropDivorced", "PropLtHighSchool", "PropHomeOwner", "PropNonUs",

"PropYrs6574", "PropWidowed", "PropHWhite", "MedianHhIncome",

"PropYrs4554")

censusvars = c("PropMarried", "PropYrs85Plus",

"PropBlack", "PropYrs519", "PerCapitaIncome", "PropNhWhite",

"PropNhBlack", "PropUnemployed", "PropHBlack", "PropCollegeGrad",

"PropPoverty", "PropAsian", "PropHighSchool", "PropFoodStamps",

"PropWhite", "PropHispanic", "PropYrs3544", "PropYrs5564", "PropYrs2534",

"PropDisabled", "PropNeverMarried", "PropYrsUnder5", "PropSomeCollege",

"PropYrs2024", "PropEmployed", "PropYrs7584", "MedianEarnings",

"PropDivorced", "PropLtHighSchool", "PropHomeOwner", "PropNonUs",

"PropYrs6574", "PropWidowed", "PropHWhite", "MedianHhIncome",

"PropYrs4554")

#########################################################

#########################################################

### Posterior probability calculation ###

### Assign **class** based on maximum probability ###

### Note: additional prep **for** Table1 **package** ###

### 1) Convert all categorical variables to ###

### factors ###

### 2) Continuous variables **as** numeric ###

### 3) Pull out **number** **from** census strings ###

#########################################################

#########################################################

post3 = **as**.data.frame(poLCA.posterior(lc=lc3, y=post))

post3 = post3 %>% mutate(rownum = row_number())

post3 = merge(for_lca, post3, by="rownum")

post3 = post3 %>% mutate(maxprob = pmax(V1,V2,V3),

**class** = ifelse(V1 == maxprob, 1,

ifelse(V2 == maxprob, 2,

ifelse(V3 == maxprob, 3, 99))))

write.csv(**as**.data.frame(names(post3)),"names.csv")

post3[catvars] = lapply(post3[catvars], factor)

post3[censusvars] = lapply(post3[censusvars], parse_number,na=c("","NA","No data found","No data"))

post3[contvars] = lapply(post3[contvars],**as**.numeric)

post4 = **as**.data.frame(poLCA.posterior(lc=lc4, y=post))

post4 = post4 %>% mutate(rownum = row_number())

post4 = merge(for_lca, post4, by="rownum")

post4 = post4 %>% mutate(maxprob = pmax(V1,V2,V3,V4),

**class** = ifelse(V1 == maxprob, 1,

ifelse(V2 == maxprob, 2,

ifelse(V3 == maxprob, 3,

ifelse(V4 == maxprob, 4,99)))))

post4[catvars] = lapply(post4[catvars], factor)

post4[censusvars] = lapply(post4[censusvars], parse_number,na=c("","NA","No data found","No data"))

post4[contvars] = lapply(post4[contvars],**as**.numeric)

post5 = **as**.data.frame(poLCA.posterior(lc=lc5, y=post))

post5 = post5 %>% mutate(rownum = row_number())

post5 = merge(for_lca, post5, by="rownum")

post5 = post5 %>% mutate(maxprob = pmax(V1,V2,V3,V4,V5),

**class** = ifelse(V1 == maxprob, 1,

ifelse(V2 == maxprob, 2,

ifelse(V3 == maxprob, 3,

ifelse(V4 == maxprob, 4,

ifelse(V5 == maxprob, 5,99))))))

post5[catvars] = lapply(post5[catvars], factor)

post5[censusvars] = lapply(post5[censusvars], parse_number,na=c("","NA","No data found","No data"))

post5[contvars] = lapply(post5[contvars],**as**.numeric)

post6 = **as**.data.frame(poLCA.posterior(lc=lc6, y=post))

post6 = post6 %>% mutate(rownum = row_number())

post6 = merge(for_lca, post6, by="rownum")

post6 = post6 %>% mutate(maxprob = pmax(V1,V2,V3,V4,V5,V6),

**class** = ifelse(V1 == maxprob, 1,

ifelse(V2 == maxprob, 2,

ifelse(V3 == maxprob, 3,

ifelse(V4 == maxprob, 4,

ifelse(V5 == maxprob, 5,

ifelse(V6 == maxprob, 6, 99)))))))

post6[catvars] = lapply(post6[catvars], factor)

post6[censusvars] = lapply(post6[censusvars], parse_number,na=c("","NA","No data found","No data"))

post6[contvars] = lapply(post6[contvars],**as**.numeric)

tablevars = c("pt_age", "age", "gender", "cat_race", "raceeth", "insurance", "elix_readmit", "elix_mortality",

"CHF", "Arrhythmia", "Valvular", "PHTN", "PVD",

"HTN", "Paralysis", "flag_neuro", "Pulmonary", "DM", "DMcx",

"Hypothyroid", "Renal", "flag_liver", "PUD", "HIV", "Lymphoma", "Mets",

"Tumor", "Rheumatic", "Coagulopathy", "flag_obesity", "WeightLoss",

"FluidsLytes", "BloodLoss", "Anemia", "flag_alcohol", "flag_drugs", "flag_psychoses",

"flag_depression", "max_pain", "service_cat", "flag_chronpain", "flag_opioid",

"flag_pos_opioids", "flag_dx_opioid", "flag_given_narcan", "flag_test_urinedrug", "flag_ds_opioids",

"flag_ds_noprescribe_opioids", "flag_ds_noprescribe_benzo", "flag_ds_noprescribe_amphet",

"flag_ds_pos_opioids", "flag_ds_pos_cocaine", "flag_ds_pos_phencyc",

"flag_ds_pos_benzo", "flag_ds_pos_amphet", "cat_ip365", "cat_op365", "cat_ed365",

"PropYrsUnder5", "PropYrs519", "PropYrs2024", "PropYrs2534","PropYrs3544", "PropYrs4554", "PropYrs5564", "PropYrs6574", "PropYrs7584", "PropYrs85Plus",

"PropBlack", "PropNhWhite", "PropNhBlack","PropHWhite", "PropHBlack", "PropHispanic",

"PropMarried", "PropPoverty", "PropFoodStamps",

"PropEmployed", "MedianEarnings", "MedianHhIncome", "PerCapitaIncome",

"PropCollegeGrad", "PropHomeOwner", "PropNonUs" )

#########################################################

#########################################################

### Create Table One **for** potential LCA solutions ###

#########################################################

#########################################################

#dput(names(post))

overall = CreateTableOne(vars=tablevars, data=post3,test=FALSE, testExact = NULL, includeNA = FALSE, testNonNormal = kruskal.test)

o3 = CreateTableOne(vars=tablevars, data=post3,test=FALSE, testExact = NULL, includeNA = FALSE, testNonNormal = kruskal.test, strata = "class")

o4 = CreateTableOne(vars=tablevars, data=post4,test=FALSE, testExact = NULL, includeNA = FALSE, testNonNormal = kruskal.test, strata = "class")

o5 = CreateTableOne(vars=tablevars, data=post5,test=FALSE, testExact = NULL, includeNA = FALSE, testNonNormal = kruskal.test, strata = "class")

o6 = CreateTableOne(vars=tablevars, data=post6,test=FALSE, testExact = NULL, includeNA = FALSE, testNonNormal = kruskal.test, strata = "class")

overall = print(overall, noSpaces = TRUE)

o3 = print(o3, noSpaces = TRUE)

o4 = print(o4, noSpaces = TRUE)

o5 = print(o5, noSpaces = TRUE)

o6 = print(o6, noSpaces = TRUE)

write.csv(overall, "lca_refine_overall.csv")

write.csv(o3, "lca_refine_3class.csv")

write.csv(o4, "lca_refine_4class.csv")

write.csv(o5, "lca_refine_5class.csv")

write.csv(o6, "lca_refine_6class.csv")

post3_trim = post3 %>% mutate(year = year(admit_date)) %>% select(mrn, hsp_account_id, **class**, year)

post4_trim = post4 %>% mutate(year = year(admit_date)) %>% select(mrn, hsp_account_id, **class**, year)

post5_trim = post5 %>% mutate(year = year(admit_date)) %>% select(mrn, hsp_account_id, **class**, year)

write.csv(post3_trim, "lca_3class_assignments.csv")

write.csv(post4_trim, "lca_4class_assignments.csv")

write.csv(post5_trim, "lca_5class_assignments.csv")

table(post3$class)

table(post4$class)

table(post5$class)

table(post6$class)

#

# for_sas = for_lca %>% dplyr::select(age, sex, cat_race, cat_ins,cat_op365, cat_ed365,

# Alcohol, Drugs, Psychoses, Depression,NeuroOther, Liver, Obesity,

# flag_dx_chronpain)

# write_csv(for_sas, "/home/shared/PROJECTS/03_OpioidPhenotype/DATA/2018-12-04_analytic.csv")

#Stacked bar plot **of** classes by year

post3 %>% mutate(Year = year(admit_date)) %>%

# group_by(Year, **class**) %>% tally()

filter(Year >= 2007) %>%

ggplot(aes(x = Year, fill = factor(**class**), col = factor(**class**))) +

geom_bar(position = "fill") + ylab("Percent") + scale_x_continuous(breaks = c(2007:2017))

post4 %>% mutate(Year = year(admit_date)) %>%

mutate(Class = **as**.factor(**class**)) %>%

filter(Year >= 2007) %>%

ggplot(aes(x = Year, fill = Class, col = Class)) +

geom_bar(position = "fill") + ylab("Percent") + scale_x_continuous(breaks = c(2007:2017)) +

scale_fill_grey() + scale_colour_grey()

table(post4$class)

mean(post4$maxprob)

sd(post4$maxprob)

post4 %>% group_by(**class**) %>% summarize(mean = mean(maxprob), sd = sd(maxprob))

table(post5$class)

mean(post5$maxprob)

sd(post5$maxprob)

post5 %>% group_by(**class**) %>% summarize(mean = mean(maxprob), sd = sd(maxprob))
