# Supplementary figures and images for: Subtypes in patients with opioid misuse: A prognostic enrichment strategy using electronic health record data in hospitalized patients
Source: PLoS One. 2019 Jul 16;14(7):e0219717. doi: 10.1371/journal.pone.0219717 (PMC6634397; doi:10.1371/journal.pone.0219717)

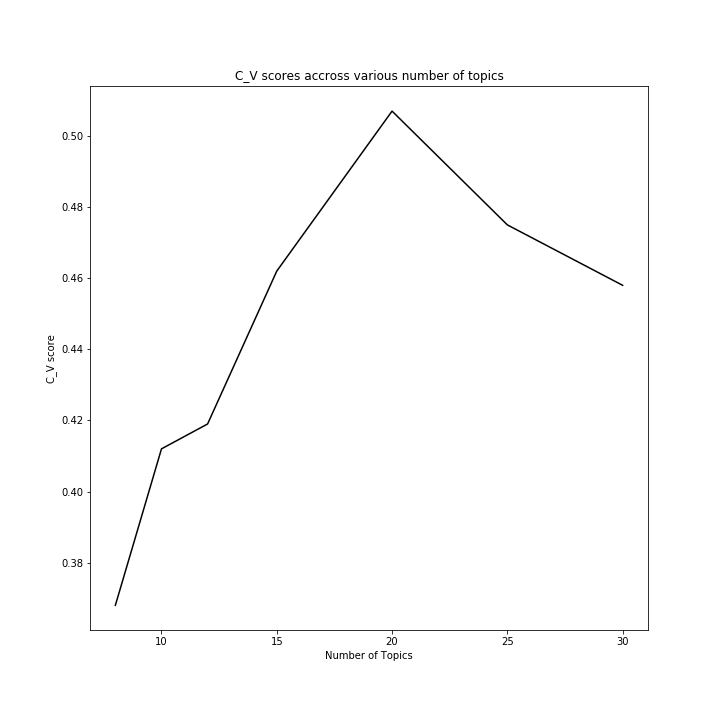

Supplement: S4 Appendix Figure — (PNG) [file pone.0219717.s004.png]
