# Supplementary material for: Subtypes in patients with opioid misuse: A prognostic enrichment strategy using electronic health record data in hospitalized patients
Source: PLoS One. 2019 Jul 16;14(7):e0219717. doi: 10.1371/journal.pone.0219717 (PMC6634397; doi:10.1371/journal.pone.0219717)
Supplement: S5 Appendix Table — (DOCX) [file pone.0219717.s005.docx]

**Supplemental 4. Participant characteristics by latent class for 5-class model**

|  | Overall  n=6224 | Class 1  n=508 | Class 2  n=396 | Class 3  n=833 | Class 4  n=2092 | Class 5  n=2395 |
| --- | --- | --- | --- | --- | --- | --- |
| Posterior probability for class, mean (SD) | 0.83 (0.14) | 0.79 (0.18) | 0.88 (0.16) | 0.87 (0.15) | 0.74 (0.10) | 0.88 (0.11) |
| Age, mean (SD) | 45.1 (14.1) | 47.8 (16.9) | 48.1 (14.6) | 43.4 (10.7) | 45.8 (15.9) | 49.1 (12.3) |
| Age†, n (%) |  |  |  |  |  |  |
| ≤ 25 | 413 (6.6) | 67 (13.2) | 33 (8.3) | 48 (5.8) | 207 (9.9) | 58 (2.4) |
| 26-35 | 1062 (17.1) | 78 (15.4) | 56 (14.1) | 171 (20.5) | 442 (21.1) | 315 (13.2) |
| 36-45 | 1209 (19.4) | 63 (12.4) | 62 (15.7) | 196 (23.5) | 380 (18.2) | 508 (21.2) |
| 46-55 | 1790 (28.8) | 116 (22.8) | 117 (29.5) | 324 (38.9) | 479 (22.9) | 754 (31.5) |
| ≥ 55 | 1750 (28.1) | 184 (36.2) | 128 (32.3) | 94 (11.3) | 584 (27.9) | 760 (31.7) |
| Male, n (%) | 3816 (61.3) | 330 (65.0) | 210 (53.0) | 491 (58.9) | 1024 (48.9) | 1761 (73.5) |
| Race, n (%) |  |  |  |  |  |  |
| Non-Hispanic Black | 2443 (39.3) | 218 (42.9) | 122 (30.8) | 480 (57.6) | 849 (40.6) | 774 (32.3) |
| Non-Hispanic White | 3037 (48.8) | 217 (42.7) | 228 (57.6) | 263 (31.6) | 1026 (49.0) | 1303 (54.4) |
| Hispanic | 571 (9.2) | 52 (10.2) | 34 (8.6) | 69 (8.3) | 177 (8.5) | 239 (10.0) |
| Other/Unknown | 173 (2.8) | 21 (4.1) | 12 (3.0) | 21 (2.5) | 40 (1.9) | 79 (3.3) |
| Insurance, n (%) |  |  |  |  |  |  |
| Medicaid | 2248 (36.1) | 162 (31.9) | 143 (36.1) | 415 (49.8) | 751 (35.9) | 777 (32.4) |
| Medicare | 1504 (24.2) | 127 (25.0) | 104 (26.3) | 95 (11.4) | 640 (30.6) | 538 (22.5) |
| Private | 1081 (17.4) | 97 (19.1) | 91 (23.0) | 59 (7.1) | 399 (19.1) | 435 (18.2) |
| Other | 1391 (22.3) | 122 (24.0) | 58 (14.6) | 264 (31.7) | 302 (14.4) | 645 (26.9) |
| Elixhauser, mean (SD) |  |  |  |  |  |  |
| Readmission score | 34.1 (18.6) | 27.3 (17.8) | 29.4 (20.1) | 32.1 (15.7) | 32.5 (17.3) | 38.5 (19.5) |
| Mortality score | 3.7 (12.5) | 4.6 (11.8) | 5.5 (12.2) | -0.4 (9.9) | 1.8 (12.3) | 6.2 (13.0) |
| Comorbidities, n (%) |  |  |  |  |  |  |
| CHF | 710 (11.4) | 57 (11.2) | 52 (13.1) | 84 (10.1) | 291 (13.9) | 226 (9.4) |
| Valvular | 729 (11.7) | 71 (14.0) | 54 (13.6) | 82 (9.8) | 265 (12.7) | 257 (10.7) |
| PHTN | 352 (5.7) | 25 (4.9) | 28 (7.1) | 38 (4.6) | 146 (7.0) | 115 (4.8) |
| PVD | 410 (6.6) | 33 (6.5) | 29 (7.3) | 32 (3.8) | 153 (7.3) | 163 (6.8) |
| HTN | 3064 (49.2) | 227 (44.7) | 203 (51.3) | 348 (41.8) | 1050 (50.2) | 1236 (51.6) |
| Paralysis | 242 (3.9) | 26 (5.1) | 15 (3.8) | 31 (3.7) | 96 (4.6) | 74 (3.1) |
| Neuro Other | 1810 (29.1) | 190 (37.4) | 136 (34.3) | 209 (25.1) | 434 (20.7) | 841 (35.1) |
| Pulmonary | 1642 (26.4) | 152 (29.9) | 107 (27.0) | 287 (34.5) | 607 (29.0) | 489 (20.4) |
| DM | 759 (12.2) | 56 (11.0) | 61 (15.4) | 74 (8.9) | 266 (12.7) | 302 (12.6) |
| DMcx | 473 (7.6) | 31 (6.1) | 32 (8.1) | 35 (4.2) | 224 (10.7) | 151 (6.3) |
| Hypothyroid | 315 (5.1) | 21 (4.1) | 23 (5.8) | 22 (2.6) | 142 (6.8) | 107 (4.5) |
| Renal | 714 (11.5) | 63 (12.4) | 29 (7.3) | 68 (8.2) | 296 (14.1) | 258 (10.8) |
| Liver† | 1180 (19.0) | 41 (8.1) | 60 (15.2) | 62 (7.4) | 11 (0.5) | 1006 (42.0) |
| PUD | 31 (0.5) | 2 (0.4) | 2 (0.5) | 2 (0.2) | 14 (0.7) | 11 (0.5) |
| HIV | 98 (1.6) | 4 (0.8) | 2 (0.5) | 13 (1.6) | 34 (1.6) | 45 (1.9) |
| Lymphoma | 87 (1.4) | 3 (0.6) | 11 (2.8) | 6 (0.7) | 49 (2.3) | 18 (0.8) |
| Mets | 238 (3.8) | 12 (2.4) | 29 (7.3) | 10 (1.2) | 124 (5.9) | 63 (2.6) |
| Tumor | 271 (4.4) | 12 (2.4) | 15 (3.8) | 18 (2.2) | 97 (4.6) | 129 (5.4) |
| Rheumatic | 136 (2.2) | 8 (1.6) | 19 (4.8) | 9 (1.1) | 76 (3.6) | 24 (1.0) |
| Coagulopathy | 762 (12.2) | 44 (8.7) | 33 (8.3) | 44 (5.3) | 140 (6.7) | 501 (20.9) |
| Obesity | 475 (7.6) | 27 (5.3) | 39 (9.8) | 37 (4.4) | 236 (11.3) | 136 (5.7) |
| Weight Loss | 557 (8.9) | 15 (3.0) | 27 (6.8) | 51 (6.1) | 204 (9.8) | 260 (10.9) |
| Fluids/Lytes | 2252 (36.2) | 183 (36.0) | 143 (36.1) | 248 (29.8) | 671 (32.1) | 1007 (42.0) |
| Blood Loss | 88 (1.4) | 8 (1.6) | 6 (1.5) | 8 (1.0) | 44 (2.1) | 22 (0.9) |
| Anemia | 1345 (21.6) | 69 (13.6) | 88 (22.2) | 121 (14.5) | 537 (25.7) | 530 (22.1) |
| Alcohol misuse† | 2421 (38.9) | 50 (9.8) | 76 (19.2) | 269 (32.3) | 0 (0.0) | 2026 (84.6) |
| Drug use | 4208 (67.6) | 281 (55.3) | 139 (35.1) | 745 (89.4) | 1695 (81.0) | 1348 (56.3) |
| Psychoses† | 1224 (19.7) | 79 (15.6) | 104 (26.3) | 209 (25.1) | 320 (15.3) | 512 (21.4) |
| Depression† | 1465 (23.5) | 80 (15.7) | 105 (26.5) | 136 (16.3) | 469 (22.4) | 675 (28.2) |
| Chronic pain† | 1829 (29.4) | 121 (23.8) | 187 (47.2) | 234 (28.1) | 696 (33.3) | 591 (24.7) |
| Opioid misuse† | 5528 (88.8) | 273 (53.7) | 126 (31.8) | 642 (77.1) | 2092 (100.0) | 2395 (100.0) |
| Service, n (%) |  |  |  |  |  |  |
| ER | 3265 (52.5) | 282 (55.5) | 232 (58.6) | 497 (59.7) | 946 (45.2) | 1308 (54.6) |
| Medicine | 1576 (25.3) | 92 (18.1) | 75 (18.9) | 165 (19.8) | 581 (27.8) | 663 (27.7) |
| Trauma | 495 (8.0) | 87 (17.1) | 54 (13.6) | 120 (14.4) | 76 (3.6) | 158 (6.6) |
| Surgery | 284 (4.6) | 6 (1.2) | 6 (1.5) | 5 (0.6) | 154 (7.4) | 113 (4.7) |
| Neurology | 166 (2.7) | 14 (2.8) | 8 (2.0) | 17 (2.0) | 61 (2.9) | 66 (2.8) |
| Other | 438 (7.0) | 27 (5.3) | 21 (5.3) | 29 (3.5) | 274 (13.1) | 87 (3.6) |
| Encounter information, n (%) |  |  |  |  |  |  |
| Given naloxone | 446 (7.2) | 70 (13.8) | 41 (10.4) | 85 (10.2) | 145 (6.9) | 105 (4.4) |
| Given a urine drug screen | 3634 (58.4) | 508 (100.0) | 396 (100.0) | 833 (100.0) | 563 (26.9) | 1334 (55.7) |
| Urine drug screen positive |  |  |  |  |  |  |
| Opioids (not given / on MAR)† | 903 (14.5) | 508 (100.0) | 42 (10.6) | 298 (35.8) | 0 (0.0) | 55 (2.3) |
| Cocaine† | 887 (14.3) | 0 (0.0) | 0 (0.0) | 794 (95.3) | 0 (0.0) | 93 (3.9) |
| Phencyclidine | 110 (1.8) | 11 (2.2) | 5 (1.3) | 34 (4.1) | 34 (1.6) | 26 (1.1) |
| Benzodiazepines (not given / on MAR)† | 631 (10.1) | 83 (16.3) | 394 (99.5) | 151 (18.1) | 0 (0.0) | 3 (0.1) |
| Amphetamines (not given / on MAR) | 107 (1.7) | 24 (4.7) | 15 (3.8) | 32 (3.8) | 6 (0.3) | 2 (0.1) |
| Prior encounters (1 year), n (%) |  |  |  |  |  |  |
| Outpatient |  |  |  |  |  |  |
| ≥ 3 | 1644 (26.4) | 66 (13.0) | 123 (31.1) | 77 (9.2) | 833 (39.8) | 545 (22.8) |
| 1-2 | 765 (12.3) | 38 (7.5) | 60 (15.2) | 78 (9.4) | 307 (14.7) | 282 (11.8) |
| 0 | 3815 (61.3) | 404 (79.5) | 213 (53.8) | 678 (81.4) | 952 (45.5) | 1568 (65.5) |
| Any ED | 1914 (30.8) | 100 (19.7) | 128 (32.3) | 255 (30.6) | 761 (36.4) | 670 (28.0) |
| Any IP | 2433 (39.1) | 106 (20.9) | 163 (41.2) | 235 (28.2) | 1016 (48.6) | 913 (38.1) |
| Census tract, mean (SD) |  |  |  |  |  |  |
| % Poverty | 12.9 (11.4) | 12.8 (11.0) | 11.7 (10.2) | 16.0 (12.3) | 13.0 (11.9) | 12.1 (10.9) |
| % Employed | 38.1 (18.5) | 37.2 (18.6) | 38.9 (18.1) | 37.8 (16.8) | 38.6 (18.6) | 37.9 (18.9) |
| Median household income ($) | 47351 (30037) | 46464 (30729) | 49820 (29955) | 43067 (25253) | 48353 (30262) | 47746 (31099) |
| % College graduate | 14.00 (9.4) | 13.4 (9.3) | 15.1 (9.6) | 12.5 (8.2) | 14.4 (9.6) | 14.1 (9.5) |
| % Home owner | 47.6 (23.9) | 46.5 (23.9) | 49.7 (24.1) | 46.4 (21.2) | 48.3 (24.0) | 47.2 (24.6) |

†indicates variable was included in LCA
