# Supplementary material for: Subtypes in patients with opioid misuse: A prognostic enrichment strategy using electronic health record data in hospitalized patients
Source: PLoS One. 2019 Jul 16;14(7):e0219717. doi: 10.1371/journal.pone.0219717 (PMC6634397; doi:10.1371/journal.pone.0219717)
Supplement: S6 Appendix Figure/Table — (DOCX) [file pone.0219717.s006.docx]

**SUPPLEMENTAL 5. Coherence plot and characteristics of sensitivity analysis at patient-level**


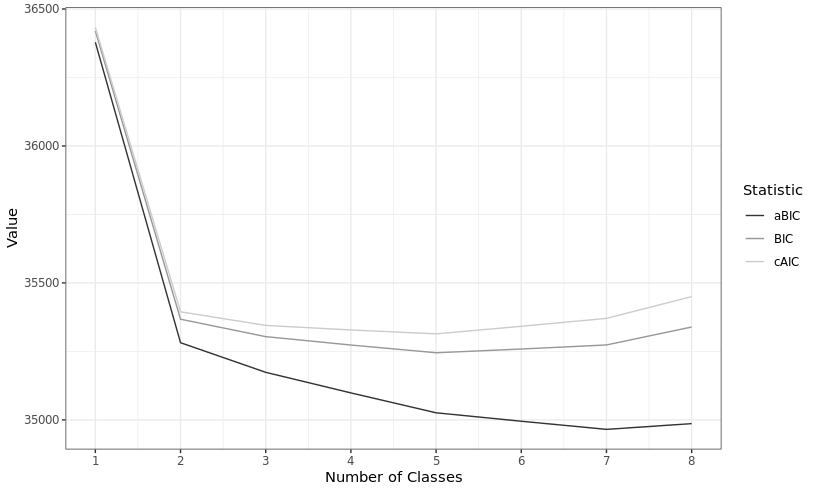


|  | **Class 1** High hospital utilization with known opioid-related conditions (n=1254) | **Class 2** Illicit use, low SES, and psychoses (n=474)) | **Class 3** Alcohol use disorders with complications (n=880) | **Class 4** Low hospital utilization and incidental opioid misuse (n=462) |
| --- | --- | --- | --- | --- |
| Probability Assignment, mean (sd) | 0.92 (0.12) | 0.91 (0.12) | 0.77 (0.11) | 0.94 (0.14) |
| age (%) |  |  |  |  |
| ≤25 | 118 (9.4) | 31 (6.5) | 22 (2.5) | 76 (16.5) |
| 26-35 | 220 (17.5) | 98 (20.7) | 128 (14.5) | 76 (16.5) |
| 36-45 | 219 (17.5) | 112 (23.6) | 207 (23.5) | 66 (14.3) |
| 46-55 | 267 (21.3) | 162 (34.2) | 272 (30.9) | 118 (25.5) |
| ≥55 | 430 (34.3) | 71 (15.0) | 251 (28.5) | 126 (27.3) |
| Male, n (%) | 707 (56.4) | 303 (63.9) | 664 (75.5) | 263 (56.9) |
| Race/Ethnicity, n (%) | | |  |  |
| Non-Hispanic Black | 444 (35.4) | 250 (52.7) | 228 (25.9) | 135 (29.2) |
| Non-Hispanic White | 666 (53.1) | 171 (36.1) | 523 (59.4) | 250 (54.1) |
| Hispanic | 107 (8.5) | 39 (8.2) | 98 (11.1) | 57 (12.3) |
| Other/Unknown | 37 (3.0) | 14 (3.0) | 31 (3.5) | 20 (4.3) |
| Insurance, n (%) | |  |  |  |
| Other | 260 (20.7) | 166 (35.0) | 268 (30.5) | 95 (20.6) |
| Private | 252 (20.1) | 37 (7.8) | 197 (22.4) | 108 (23.4) |
| Medicaid | 416 (33.2) | 225 (47.5) | 252 (28.6) | 164 (35.5) |
| Medicare | 326 (26.0) | 46 (9.7) | 163 (18.5) | 95 (20.6) |
| Elixhauser Mortality score, mean (SD) | 3.45 (13.13) | -1.59 (9.95) | 5.79 (13.67) | 4.03 (11.46) |
| Comorbidities, n (%) | | |  |  |
| CHF | 157 (12.5) | 34 (7.2) | 64 (7.3) | 44 (9.5) |
| Hypertension | 591 (47.1) | 175 (36.9) | 408 (46.4) | 186 (40.3) |
| Neuro | 303 (24.2) | 118 (24.9) | 314 (35.7) | 166 (35.9) |
| Pulmonary | 348 (27.8) | 131 (27.6) | 147 (16.7) | 112 (24.2) |
| Diabetes Mellitus | 136 (10.8) | 36 (7.6) | 85 (9.7) | 48 (10.4) |
| Renal | 141 (11.2) | 26 (5.5) | 66 (7.5) | 35 (7.6) |
| Liver† | 169 (13.5) | 25 (5.3) | 259 (29.4) | 44 (9.5) |
| HIV | 20 (1.6) | 5 (1.1) | 13 (1.5) | 3 (0.6) |
| Alcohol use disorder† | 35 (2.8) | 174 (36.7) | 880 (100.0) | 79 (17.1) |
| Drug use | 984 (78.5) | 438 (92.4) | 442 (50.2) | 178 (38.5) |
| Psychoses† | 181 (14.4) | 119 (25.1) | 210 (23.9) | 106 (22.9) |
| Depression† | 252 (20.1) | 85 (17.9) | 222 (25.2) | 102 (22.1) |
| Chronic pain† | 356 (28.4) | 138 (29.1) | 195 (22.2) | 176 (38.1) |
| Opioid misuse† | 1254 (100.0) | 380 (80.2) | 880 (100.0) | 162 (35.1) |
| Service, n (%) | |  |  |  |
| ER | 536 (42.7) | 271 (57.2) | 444 (50.5) | 245 (53.0) |
| MEDICINE | 314 (25.0) | 77 (16.2) | 244 (27.7) | 66 (14.3) |
| NEUROLOGY | 51 (4.1) | 9 (1.9) | 32 (3.6) | 13 (2.8) |
| OTHER | 163 (13.0) | 22 (4.6) | 38 (4.3) | 29 (6.3) |
| SURGERY | 99 (7.9) | 3 (0.6) | 37 (4.2) | 4 (0.9) |
| TRAUMA | 91 (7.3) | 92 (19.4) | 85 (9.7) | 105 (22.7) |
| Encounters, n (%) | |  |  |  |
| Given naloxone | 124 (9.9) | 54 (11.4) | 38 (4.3) | 61 (13.2) |
| Given a urine drug screen | 457 (36.4) | 474 (100.0) | 533 (60.6) | 462 (100.0) |
| Urine drug screen (+) | | |  |  |
| Opioids (not given / on MAR)† | 98 (7.8) | 178 (37.6) | 15 (1.7) | 268 (58.0) |
| Benzodiazepines† | 0 (0.0) | 85 (17.9) | 0 (0.0) | 277 (60.0) |
| Amphetamines | 7 (0.6) | 8 (1.7) | 1 (0.1) | 33 (7.1) |
| Cocaine† | 0 (0.0) | 474 (100.0) | 9 (1.0) | 1 (0.2) |
| Phencyclidine | 27 (2.2) | 22 (4.6) | 15 (1.7) | 18 (3.9) |
| Amphetamines | 18 (1.4) | 11 (2.3) | 6 (0.7) | 33 (7.1) |
| Prior encounters (1 year), n (%) | | | |  |
| Outpatient | |  |  |  |
| 0 | 709 (56.5) | 421 (88.8) | 681 (77.4) | 360 (77.9) |
| 1-2 | 161 (12.8) | 32 (6.8) | 92 (10.5) | 38 (8.2) |
| ≥ 3 | 384 (30.6) | 21 (4.4) | 107 (12.2) | 64 (13.9) |
| Any ED | 267 (21.3) | 106 (22.4) | 150 (17.0) | 77 (16.7) |
| Census tract, mean (SD) | | |  |  |
| % High Poverty | 12.85 (11.74) | 15.04 (12.41) | 11.74 (10.99) | 12.01 (11.18) |
| % Employed | 38.45 (18.71) | 37.10 (17.79) | 37.01 (19.78) | 38.02 (19.04) |
| Household earnings   (Median $) | 48300.16 (30866.01) | 42854.38 (26988.79) | 46613.39 (32505.06) | 49654.01 (33083.35) |
| % College graduate | 14.51 (9.91) | 12.71 (8.60) | 13.72 (9.68) | 14.82 (9.93) |
| % Home owner | 48.34 (24.22) | 45.71 (22.29) | 45.72 (25.36) | 48.07 (24.78) |

†indicates variable was included in LCA. For benzodiazepines and amphetamines, the drug was not administered during hospital encounter prior to urine drug screen testing and not on the patient’s medication administration record.

*Drug abuse codes include drug-induced mental disorder, psychoactive substances, drug-persisting dementia, polysubstance use, and use during pregnancy.

High-poverty census-tract (20.0+ percent of households below federal poverty level)
